# Variable structure motifs for transcription factor binding sites

**DOI:** 10.1186/1471-2164-11-30

**Published:** 2010-01-14

**Authors:** John E Reid, Kenneth J Evans, Nigel Dyer, Lorenz Wernisch, Sascha Ott

**Affiliations:** 1 MRC Biostatistics Unit, Institute of Public Health, Forvie Site, Robinson Way, Cambridge, CB2 0SR, UK; 2 School of Crystallography, Birkbeck College, Malet Street, London, WC1E 7HX, UK; 3 MOAC Doctoral Training Centre, Coventry House, University of Warwick, Coventry, CV4 7AL, UK; 4 Systems Biology Centre, Coventry House, University of Warwick, Coventry, CV4 7AL, UK

## Abstract

**Background:**

Classically, models of DNA-transcription factor binding sites (TFBSs) have been based on relatively few known instances and have treated them as sites of fixed length using position weight matrices (PWMs). Various extensions to this model have been proposed, most of which take account of dependencies between the bases in the binding sites. However, some transcription factors are known to exhibit some flexibility and bind to DNA in more than one possible physical configuration. In some cases this variation is known to affect the function of binding sites. With the increasing volume of ChIP-seq data available it is now possible to investigate models that incorporate this flexibility. Previous work on variable length models has been constrained by: a focus on specific zinc finger proteins in yeast using restrictive models; a reliance on hand-crafted models for just one transcription factor at a time; and a lack of evaluation on realistically sized data sets.

**Results:**

We re-analysed binding sites from the TRANSFAC database and found motivating examples where our new variable length model provides a better fit. We analysed several ChIP-seq data sets with a novel motif search algorithm and compared the results to one of the best standard PWM finders and a recently developed alternative method for finding motifs of variable structure. All the methods performed comparably in held-out cross validation tests. Known motifs of variable structure were recovered for p53, Stat5a and Stat5b. In addition our method recovered a novel generalised version of an existing PWM for Sp1 that allows for variable length binding. This motif improved classification performance.

**Conclusions:**

We have presented a new gapped PWM model for variable length DNA binding sites that is not too restrictive nor over-parameterised. Our comparison with existing tools shows that on average it does not have better predictive accuracy than existing methods. However, it does provide more interpretable models of motifs of variable structure that are suitable for follow-up structural studies. To our knowledge, we are the first to apply variable length motif models to eukaryotic ChIP-seq data sets and consequently the first to show their value in this domain. The results include a novel motif for the ubiquitous transcription factor Sp1.

## Background

This paper examines the problem of modelling and discovering sequence motifs for transcription factors that exhibit flexible DNA binding preferences.

### Modelling binding sites

Transcriptional regulation is an important part of regulatory control in eukaryotes. Experimental techniques to determine which transcription factors bind which loci in particular cell types under specific conditions are improving at a rapid rate. However, we are a long way from determining the binding sites of all transcription factors in all conditions. Until we have this experimental data, mathematical models of binding sites will help us predict TFBSs and in turn help us infer regulatory effects. These models may reveal combined binding sites of a transcription factor and its co-factors [1] and can be used to identify binding sites in species for which experimental binding data is not available. Furthermore, such models can explain variation in binding affinities [2,3] that can have a functional effect. Therefore, building such models is a crucial task in current bioinformatics research.

Traditionally models of TFBSs have been of fixed width. These PWMs model each position of a binding site independently. By using motifs of fixed length, these methods implicitly assume proteins bind to different sites in the same structural configuration. However, some protein-DNA interactions exhibit more flexibility and bind their target regions in different configurations resulting in binding sites of different widths. For example, the Pit-1 homodimer is known to accommodate flexible spacing between its half-sites [4]. The function of the two binding configurations differs through the interaction of co-factors: one acts as a repressor; the other as an activator. Other transcription factors are known to accommodate variable length binding sites for their DNA interactions, for example, p53 [5] and the Stat family of proteins [6,7]. Variable spacers in p53 binding sites have been shown to increase the binding affinity 6.6-fold [8]. Publicly available ChIP-seq data providing thousands of experimentally verified binding regions make it possible to search for other examples of variable width binding preferences. Transcription factors that have such binding preferences may be in the minority.

Until recently the data upon which models of binding sites have been either obtained from rather artificial *in vitro *experiments, for example, SELEX [9], or from painstakingly collected single *in vivo *binding sites. Such binding sites and models of transcription factor binding preferences have been compiled in databases such as TRANSFAC [10] and JASPAR [11]. In the Results section we present an examination of the binding sites in TRANSFAC that suggests variable length binding site models may be useful. However, the number of binding sites for most transcription factors modelled in TRANSFAC is fairly low and this limits the conclusions that can be drawn from this analysis. The increasing availability of data from high-throughput ChIP assays enables us to investigate more complex models of transcription factor binding preferences.

### Motif search

New techniques such as ChIP-chip, ChIP-PET, or ChIP-seq are providing large volumes of genome-wide data on regions of transcription factor binding [1,12-17]. While the identification of genomic target regions from these data is straightforward, motif search techniques are still required to identify the exact binding positions and to learn mathematical models of transcription factor binding. Motif search is a notoriously difficult problem: Harbison et al. [18] found that significant results were reported in randomly generated data sets.

A host of motif finding techniques are available. A large subset of motif-finders such as MEME [19], NMica [20], AlignACE [21] or MDscan [22] fit PWMs to the sequence data. Reviews of the sensitivity and specificity of these methods include [23] and [24]. Discriminative techniques that explain the ranking of fold changes have recently made an impact [25,26]. Methods that make use of 3D structures of transcription factors binding DNA oligos to inform prior probability distributions have been proposed [27]. Existing variations of the weight matrix model include specialisations such as consensus sequences [28] or palindromic weight matrices [29], and also generalisations such as models that allow for dependencies between non-neighbouring bases [30] or models of dimers binding to two half-sites that feature certain spacing rules [31,32]. These extensions are placed in a formal framework by Brazma et al. [33].

### Variable length models and search

van Helden et al. consider a model of spaced dyads, where two words of length three are separated by a spacer of a fixed length [31]. The spacer has no preference for particular nucleotides and typically has a length between 0 and 16 bases. No degeneracy is allowed in the words. The reported dyads (motifs) incorporate no variability in their spacer lengths but a range of values are tested during the search for the best dyads. The approach is designed to detect binding sites for *C*_6_*Zn*_2 _binuclear cluster proteins in yeast. The authors discuss that other organisms typically have a higher degree of degeneracy in the binding sites for their transcription factors and that perhaps their method is best suited for yeast.

Carvalho et al. [32] present an exact method, RISO, to detect *structured motifs*. A structured motif is a set of words with user specified spacing rules. RISO can be seen as an extension of the work of van Helden et al. in two directions: whilst the motif model contains no degeneracy itself, mismatches are allowed in the sites during search; also, the resulting binding sites are allowed flexible spacing to accommodate variable length motifs. RISO uses a truncated generalised suffix tree for efficient enumeration during its search for motifs. The application and results focus on zinc cluster transcription factors in yeast.

Frith et al. have developed a method, GLAM2 [4], to find motifs with arbitrary insertions and deletions. They mainly apply it to protein sequences although one application to short (31 base pair) DNA sequences is presented. Allowing arbitrary insertions and deletions increases the number of parameters of the model considerably. To the best of our knowledge it has not been used to find variable length motifs in data sets of the size that ChIP-seq generates.

A recent review of transcriptional control by p53 in humans [5] highlights the ability of the p53 protein to bind sites of variable length. In another work [35], a profile hidden Markov Model is hand-crafted to model insertions and deletions in a set of known binding sites. The task of learning a motif from ChIP-seq data is not addressed.

Previous work on the Stat family of proteins [6,7] has highlighted their ability to bind to variable length binding sites. In particular, Soldaini et al. [6] examine the spacing between two Stat5a homodimers when bound as a tetramer. They hypothesise that the variable spacing may influence the degree of Stat-mediated DNA bending and hence have an important functional effect of the transcriptional activation of Stat5a target genes. They also suggest the variable spacing may be a mechanism to control which co-factors interact with Stat5a. Ehret et al. [7] examine spacing rules in Stat1, Stat5 and Stat6 homodimer binding sites. They use a hand-crafted hidden Markov model (HMM) to learn variable length motifs for these proteins using data from *in vitro *binding site selection experiments.

Finally, Badis et al. have used protein binding arrays [36] to challenge common preconceptions about how transcription factors interact with their DNA binding sites. Their work shows that the sequence binding preferences of many proteins exhibit "secondary motifs", "position interdependence" or "variable spacer lengths". In particular, they highlight the binding preferences of Jundm2, a protein which seems well suited to a variable length model.

### Our model

**Figure 1:**
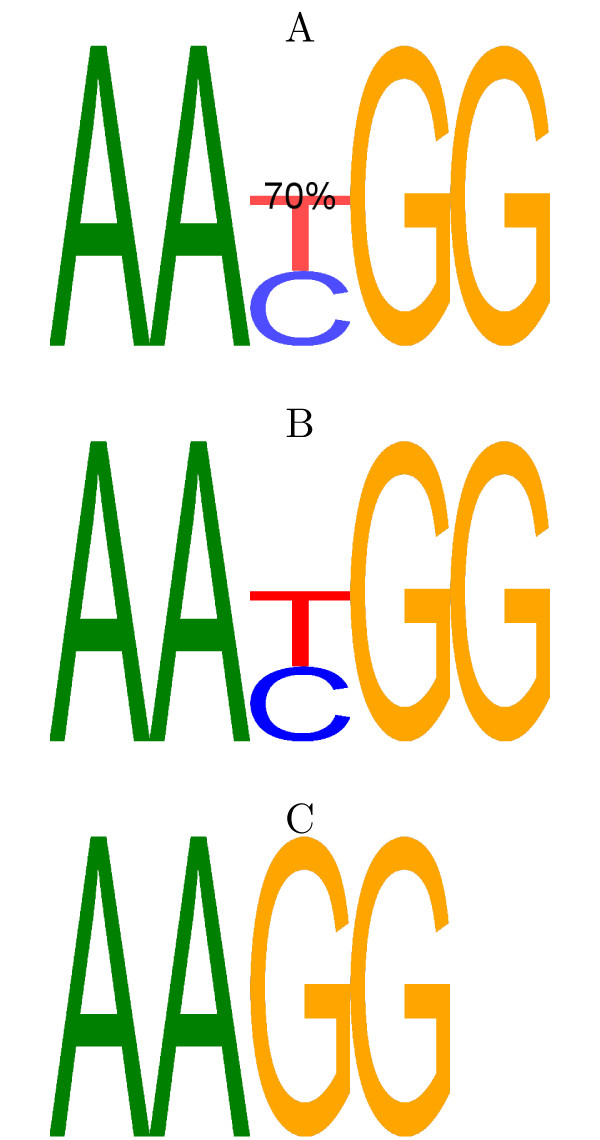
**Example gapped PWM logo**. An example to demonstrate the gapped PWM model and logo format: A gapped PWM, A, and 2 standard PWMs, B and C, are shown. All three define distributions over 5-mers: note that the last base of C is non-specific and not represented in the logo as it has no information content. The gapped PWM, A, can be viewed as a 70/30 mixture of B and C. That is, 70% of its binding sites look like sites from B and 30% look like sites from C. Put another way: 70% of its sites have a T/C inserted in the centre. The probability of the optional base being inserted in any given binding site is represented in 2 ways: firstly as a percentage written directly onto the logo; secondly, the base is also faded to represent how often it is present.

In this work we develop a general model of transcription factor binding that incorporates such variability. Our model extends the PWM model by introducing an optional character (or gap-character) to model variable-length motifs. The gap-character may appear at a certain position inside the motif with a certain frequency and it has its own nucleotide frequencies. An example of our model is shown in Figure 1. We call motifs of this form *gapped PWMs*.

A popular statistic for the information content and significance of standard PWMs is the relative entropy [37], *I*_seq_, measured against a genomic background distribution. Calculating the relative entropy is straightforward for binding site motifs that treat their positions independently. Unfortunately, the position independence assumption does not hold when gaps are introduced. It is still possible to calculate *I*_seq _for gapped PWMs but it involves an enumeration over all possible words under the motif. Thus an exact calculation is prohibitive for long motifs. We describe the calculation and show some examples highlighting the issues in the Methods section.

In the Results section we present an analysis of the binding sites in the TRANSFAC database and a comparison of our method to several others: MEME, one of the most successful and popular standard motif finders; GLAM2, the best variable length motif finder known to us; and our own method but with the possibility of a gap switched off. We compare the motifs each method finds on several data sets and perform a cross-validation test with held-out test data to analyse the predictive abilities of the methods. We show two novel motifs, one discovered by GLAM2 and one discovered by our method that may bear further investigation. The discussion section reviews the results and compares the merits and shortcomings of each of the methods. Further discussion points include the difficulty of selecting negative control sequences for testing motif finders, the possible structural reasons for variations in binding site widths, and how far allowing just one optional gap character is a limitation. We also relate the relevance of the work to databases of binding preferences determined by protein binding microarrays.

## Methods

For motif discovery our model is restricted to motifs that vary in length by one base at most. We target gapped PWMs that have a optional base near their centres. We did not allow more than one optional base as inference becomes increasingly difficult as the hypothesis space grows. In a similar spirit to the popular motif finder MDscan [22], we combine a maximum likelihood approach with enumerative methods to initialise the model's parameters. Using the Baum-Welch algorithm [38] we learn the parameters of a hidden HMM that models the background sequence and binding sites on both strands of the DNA. We use the Baum-Welch algorithm as it is the most popular technique for learning the parameters of HMMs and is guaranteed to converge to a local maximum. Viterbi training [38] and Gibbs sampling [39] are possible alternative inference techniques. Viterbi training is less popular than the Baum-Welch algorithm and is not guaranteed to converge. Gibbs sampling has been successful in several other TFBS search algorithms [40-46] but is not normally used in conjunction with HMMs. The Baum-Welch algorithm is an expectation-maximisation (EM) algorithm. EM methods have been successful in this field [22,47]. Hence we had no compelling reason to use Gibbs sampling over the Baum-Welch algorithm.

**Figure 2:**
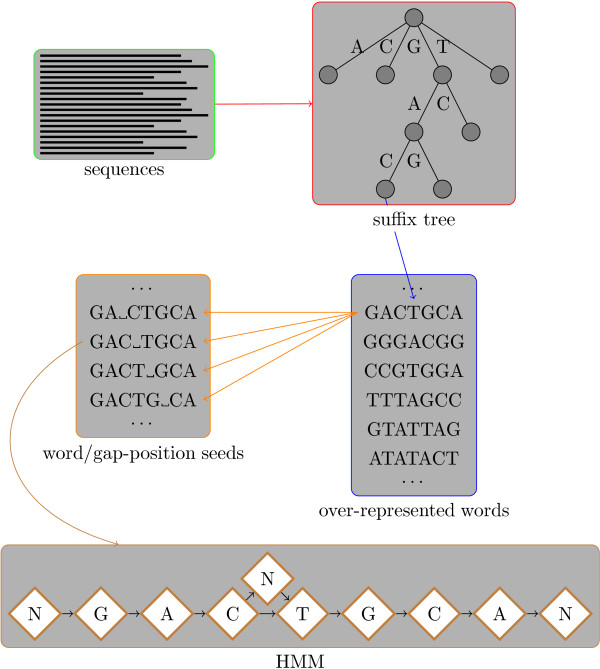
**Search method overview**. Overview of search method. The input sequences are converted into a suffix tree which is used to efficiently enumerate over-represented words. These words are tested as possible seeds for a HMM. For each seed we consider a number of different placements of the gap character. Highly scoring seeds are used to initialise HMMs which are trained using the Baum-Welch algorithm. Each trained HMM defines a gapped PWM and these are scored and ranked. The best gapped PWMs are reported as the output of the method.

Our method comprises the following stages (see Figure 2):

• Build a suffix tree representing the sequences.

• Find over-represented words in the sequences.

• Test over-represented words together with possible gap positions as candidate seeds for the HMM.

• Train HMM using the most promising seeds.

• Score, rank and filter learnt gapped PWMs.

### Finding over-represented words

Unfortunately, in the context of our problem the Baum-Welch algorithm is extremely sensitive to initial conditions. Therefore, we devote some effort to finding several good candidate initial conditions or seeds for the HMM's emission parameters.

We use a suffix tree [48] to enumerate all the *L*-mers in the sequences allowing for reverse complements (*L *is a user-specified parameter which defaults to 8). For each *L*-mer, we count how many sequences it occurs in and the number of times it occurs across all the sequences. The *L*-mers are sorted to determine which are over-represented. The primary sort key is the number of sequences the *L*-mer occurs in and the secondary sort key is the total number of occurrences across all the sequences.

Seeds that are close in edit distance to each other are likely to converge to the same gapped PWM when the Baum-Welch algorithm is applied. Therefore, we filter the *L*-mers using their edit distance from higher ranked *L*-mers. Our edit distance allows for reverse complements and shifts in the *L*-mers. Any *L*-mers that are less than a user-specified edit distance from a previously evaluated *L*-mer are discarded. The remaining *L*-mers become our candidate seeds. It should be emphasised that finding over-represented words is a heuristic for seeding the HMM only and has no influence on the final scoring by the HMM.

In addition to the *L*-mer we need to choose where to place the optional base in the PWM in order to seed the HMM. For each candidate *L*-mer we examine each possible gap position in turn. We do not allow gap positions close to the end of the motif. The first gap is allowed after the base at position ⌈*L*/5⌉ + 1 and the last gap is positioned symmetrically at the end of the motif. Each (*L*-mer, gap position) pair is scored as follows:

• We generate 2 standard PWMs from the *L*-mer: one represents binding sites which include the optional base at the given gap position, the other represents sites without the optional base. The PWM without the optional base is given an extra base at the end so both have the same length, *L *+ 1. The user specifies a pseudo-count to smooth both PWMs' distributions and the gap position is given a uniform distribution as is the extra padding base.

• We calculate the log likelihood of each *L *+ 1-mer in every sequence under a background model.

• We score each *L *+ 1-mer with both PWMs, calculating the log likelihood for both strands.

• For each *L *+ 1-mer we calculate the log likelihood ratio between the better PWM (in either orientation) and the background model.

• Each sequence is scored as the maximum of its *L *+ 1-mers' log likelihood ratios. That is, we are looking for the single best binding site on either strand of each sequence explained either by the gapped or ungapped PWM.

• The overall score for the given (*L*-mer, gap position) pair is the sum of the scores for each sequence. For each sequence we take the maximum ratio over all positions, both strands and both PWMs. The score for the seed is the sum of these maxima over all sequences.

Our scheme is motivated by a desire not to use seeds that are easily explained by a background model and to find seeds that explain sites in as many sequences as possible.

In the above scheme the background is modelled by a HMM with 4 states and Markov order 3. That is, its emissions are conditioned on the previous three observations which gives each state 256 emission parameters. The parameters for transitions out of any given state are tied. The HMM is trained on the input sequences.

### Initialisation and training of the HMM

**Figure 3:**
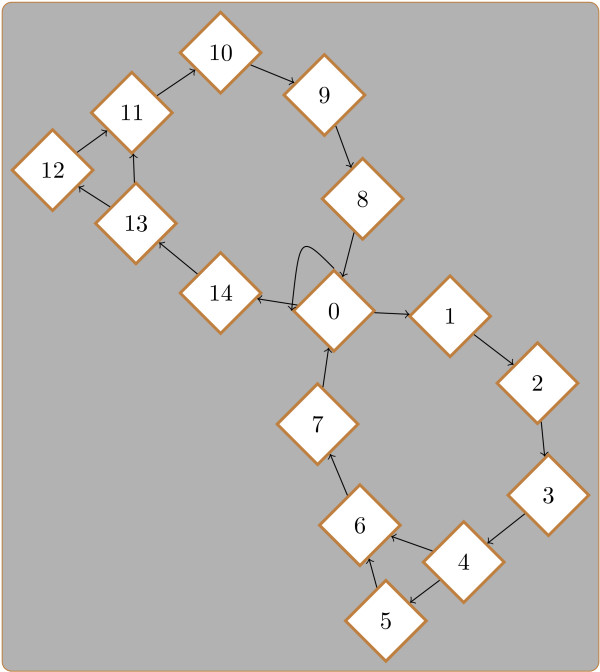
**HMM state transitions**. An example of a typical HMM state transition diagram. This HMM jointly models background sequence and binding sites from a gapped PWM of length 7. State 0 is the background state. The two arms leading out from state 0 generate binding sites on the positive and negative strands. States 1 and 14 are the first states for binding sites generated in the positive and negative direction respectively. Similarly states 5 and 12 represent the optional base for binding sites generated in the positive and negative direction respectively. When training the HMM various parameters are tied so that they are always equal. For example, the transition parameter from state 0 to state 1 is tied to the parameter for the equivalent transition to state 14. This ensures binding sites are equally likely on both strands of DNA. Similarly emission parameters are tied to ensure binding sites on the negative strand have a distribution that is the reverse complement of the distribution of the binding sites on the positive strand.

A HMM is a Markov process with unobserved states. These states can be regarded as an underlying process that generates the data. We model background genomic sequence by one set of states and binding sites by another set. Binding sites on the positive strand are generated by a distinct set of states to those on the negative strand. The HMM is parameterised by the transition probabilities between the states and the token emission probabilities for each state. We use the transition probabilities to model the relative scarcity of binding sites. In our context, the output tokens of the HMM are the nucleotide bases of the sequences. Each base in our input sequences is associated with an unobserved state. An example state transition diagram is shown in Figure 3.

For each of the highest scoring seeds (*L*-mer, gap position pairs) we create a HMM and train it. The number of seeds used for this purpose is a tunable parameter. Our experience showed the algorithm was robust to changes in this parameter as the best seeds were invariably amongst the highest scoring. In our tests we used a value of 60. The emission parameters of the states for the positive strand are initialised by the *L*-mer (with the addition of pseudo-counts). The emission parameters of the states for the negative strand are tied to the emission parameters of the states for the positive strand so that the motif is the same irrespective of the strand the binding site is on. The state transitions are initialised to reflect the gap position. We estimate the initial probability of leaving the background state by the number of occurrences of the initialising *L*-mer divided by the number of bases in the sequences. This estimate can be scaled by a user-specified parameter. It can be reduced to encourage sharper motifs with fewer binding sites or increased for more prevalent vaguer motifs. The transition probability to the gap base is initialised to 0.5. We train the HMM using the Baum-Welch algorithm. Without a prior on the transitions out of the background state, this invariably results in an extremely vague motif: that is, the model prefers a motif of high entropy with many binding sites over a motif of low entropy with a smaller but more plausible number of occurrences. We place a very strong prior on this transition that effectively fixes it and encourages the Baum-Welch algorithm to learn motifs of higher information content.

The Baum-Welch algorithm terminates when the increase in log likelihood is smaller than some threshold. We use a threshold of .0004 per sequence. If the motif becomes vague during training, we stop training and discard the model. We measure the vagueness by the entropy per base.

### Scoring the gapped PWMs

Each seed we use to initialise the HMM results in one gapped PWM. However, we are most interested in PWMs that satisfy the following criteria:

• The PWM has high information content.

• The PWM found a binding site in a high proportion of the sequences in the data set.

• The PWM does not just model lower order features in the sequences.

For each of these properties we score each PWM between 0 and 1. The score for the information content, *S*_*ic*_, is the ratio of the PWM's information content to the maximum possible. Here we calculate the information content in a naïve position-independent sense as we cannot afford the full enumeration over all possible words as described elsewhere in the Methods section. We use an approximation where each position is treated independently but the information content of the optional base is weighted by the frequency with which it occurs. The score for the number of binding sites, *S*_*bs*_, is simply the fraction of sequences for which the PWM finds at least one binding site. In order to discount PWMs that appear to model lower-order features in the sequences (for example GC rich regions), we calculate the entropy of the first-order distribution defined by the PWM. That is, we take consecutive bases in the PWM and look at their joint distribution. We take the average of these distributions over all consecutive pairs of bases and calculate its entropy. We are looking for PWMs where this first order entropy is high (for example a PWM that represents "GCGCGCGC" would have a very low entropy). Our score, *S*_*lo*_, to discount PWMs representing these lower order features is simply the ratio of the PWM's first order entropy to the maximum possible entropy.

In order to take account of the three criteria above, we score each PWM by the geometric mean of the scores. This mean is biased using weights to make the scales of the different scores comparable. Heuristically, we found suitable weights to be 1.5, 1 and 1 for *S*_*lo*_, *S*_*ic *_and *S*_*bs *_respectively. For the data sets used in this paper, the top motifs from both methods were clearly the best. In the results presented only the top motif was used for discrimination and we only report one motif per data set in the results. This is certainly an ad hoc scoring scheme, however we found it important to integrate our prior beliefs about motifs into the scoring scheme. It was not easy to encode all these beliefs into a probabilistic model or likelihood function that we could fit or optimise. In particular, the beliefs about the lower-order features were difficult to incorporate in this way. Our ad hoc scoring scheme does capture our beliefs in a straightforward manner and was found to be effective. We found the results were robust to minor variations in the values of these parameters.

### Gapped PWMs as distributions over words

We describe the details of the distribution a gapped PWM induces over words in order to make the example in Figure 1 concrete. Suppose we have a gapped PWM of length *K *(including the optional character). We treat the gapped PWM as a model of binding sites on both the positive and negative strand of DNA. In other words, we model it as a 50/50 mixture of itself and its reverse complement. Suppose that the optional character occurs in a proportion *r *of the binding sites and that the base frequencies of the equivalent standard PWM with and without the optional character are given by  and  respectively. Note that, as in the example, the frequencies for the case where the optional character is omitted are augmented by an 'N' in the last position. Suppose furthermore that  is the complement of base *b *and that  is the *k*th position in the reversed PWM, so that  = *K*,  = *K *- 1,... Then the distribution the gapped PWM induces over words is given by

where *x *= *x*_1 _... *x*_*K *_is a word.

### Information content

Probabilistic models for transcription factor binding sites such as PWMs and gapped PWMs define distributions over words of a certain length, *K*. The information content (or information gain or Kullback-Leibler divergence, *D*_*KL*_) of such a distribution, *p*(*x*), relative to a background distribution over words, *q*(*x*), is defined as

where *X *is the set of all such words. *I*_seq _measures how different the PWM, *p*(*x*), is from the background, *q*(*x*). In information theory terminology, *I*_seq _is the average message length required to transmit a binding site of the PWM using a code optimised for the background distribution.

In the position independent case when using a 0-order background model and ignoring reverse complements (see below), the sum decomposes into sums over the probabilities of bases at each position.

**Figure 4:**
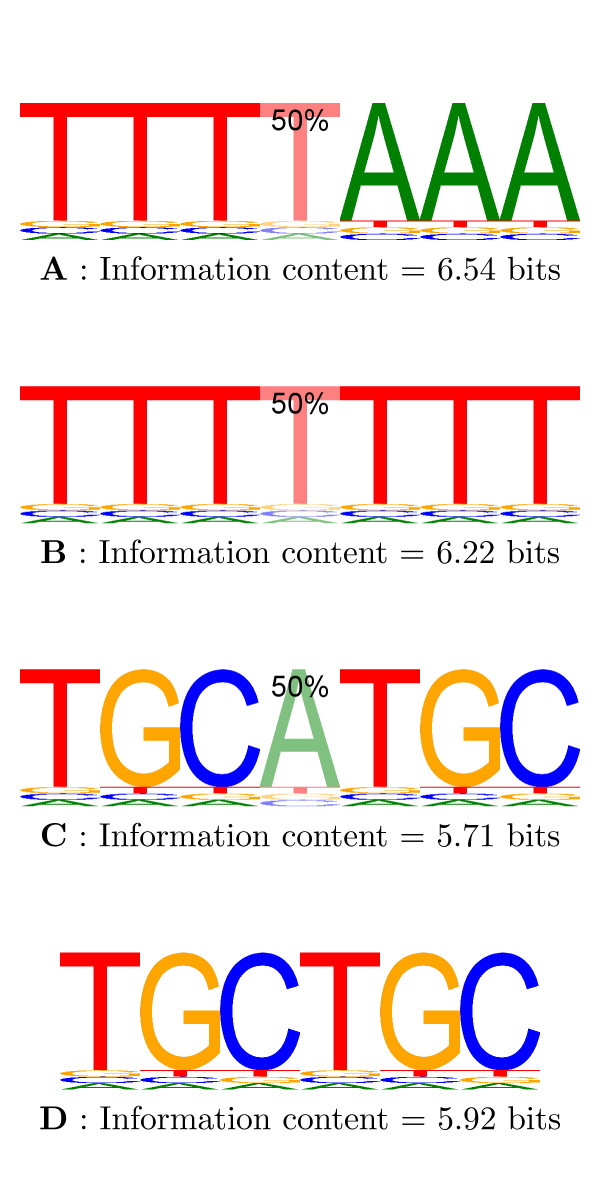
**Information content of gapped motifs**. Examples showing how position dependencies induced by gap characters can affect the information content of motifs. Compare the gapped PWM C with the standard PWM D. Here the introduction of a gap has decreased the information content as the distribution over 7-mers is more vague. In contrast, PWM B has a higher information content than PWM D. Whether the gap is present or not, the bases around it remain Ts. Hence PWM B has a much sharper distribution over 7-mers. Note the difference in information content between gapped PWMs A and B. The reason is that A is very close to its own reverse complement whereas B is not. Hence A has a sharper distribution than B. All the information contents were calculated relative to a uniform 0-order Markov model.

This leads to the well-known formula for the information content of a standard PWM to be

where *p*_*kb *_is the probability of seeing base *b *at the *k'*th position of the PWM and *q*_*b *_is the probability of base *b *in the background distribution. This decomposition ignores the fact that a PWM is almost always applied to the positive and negative strand of DNA. In light of this and continuing to view a PWM as a distribution over words, the PWM can be seen as a mixture model over two components. In one component it is applied in the positive direction. In the other component it is applied in the negative direction as a reverse complement. Put another way, which words would we score highly under the consensus sequence AACCTT? AACCTT itself of course but also its reverse complement, AAGGTT. As a PWM is almost always used in this mixture model sense, position independencies do not hold. When position independencies do not hold, the decomposition in the above sum does not hold either and in general the calculation of information content requires a sum over all words. This also applies to gapped PWMs and we show an example in Figure 4.

### Evaluation

Each method was evaluated by constructing an HMM from a single background class, (defined by single nucleotide frequencies), the motif from the method and a parameter for the transition probability from the background to the motif. For each sequence, *s*, we calculated the expected number of bases, *n*_*s*_, that have been generated by the motif under this model. For several thresholds, *t*, we calculated the proportion, *p*, of sequences (in the held out sequences from the original data set) which had *n*_*s *_>*t*, and the corresponding proportion, *q*, for a set of negative sequences. The Receiver-Operator-Characteristic (ROC) curve is the plot of *p *against *q*. The perfect ROC is the one that goes through the point (0,1), and random guessing gives the diagonal line between points (0, 0) and (1, 1). In our analyses, we used three separate negative reference sets: a) shuffled versions of the original positive sequences, b) a set of sequences taken at random from the human genome, assembly NCBI36 and c) sequences from promoter regions of randomly chosen genes starting 1000 bases upstream of the transcription start site. In each case, the sequences of the negative data set were matched in number and length to those of the positive data set. Evaluation of the motif-finders used a five-fold cross validation. The ROCs shown in the text are the accumulation of the ROCs for each of the five folds. For each ROC we calculated the area-under-the-curve (AUC) and AUC50 [49] statistics. The AUC statistic reflects the performance of the method overall and the AUC50 statistic reflects the performance of the method at high specificity. The AUC50 is the area under the ROC curve generated by discarding all but the 50 highest scoring negative examples. It is a measure of how good the method is at classifying sequences relative to the highest scoring negative examples. It is a useful metric when the user of a method can only afford to follow-up a few of the examples that they test. The details of the calculation are given in Additional file 1.

The parameters used for MEME and GLAM2 and the details of the processing of the data sets are given in Additional file 1. We should note that we used the same data sets to tune the ad hoc parameters of our method, MEME and GLAM2. This may mean that these parameters are slightly overfitted with respect to our data sets but we believe this effect is negligible. In general, we found all the methods robust to minor changes in the parameters.

### Investigation of TRANSFAC binding sites

ClustalW2 [50] release 2.0.10 was used to realign the sequences used with TRANSFAC, version 2008.3, [10] to determine the PWMs. The gap extension penalty was reduced to 7 from the default of 15, the gap extension to 3 from 6.66 and the transitions weighting to 0 from 0.5. Minor manual adjustments were made to the results to reduce the number of locations with optional gaps. The 10 PWMs where TRANSFAC had introduced gaps in order to produce their published PWMs were I$DL_01, V$MYOGNF1_01, IRF-1, V$IRF2_01, V$BRN2_01, V$ARP1_01, P$EMBP1_Q2, V$RSRFC4_Q2, V$LUN1_01, V$DEAF1_02. In all, 510 PWMs were processed through ClustalW2. Of these there were 70 PWMs where ClustalW2 introduced gaps in order to obtain alignment of one or two base-pairs on the edge. These added no significant information and were ignored. There were 58 cases where ClustalW2 introduced a gap for one site (or all but one site) in the centre of the binding region. These cases could be significant but given the small sample size, these were also ignored. There were 26 examples similar to the above where there was more than one gap that was introduced, but still there was only a single instance of each type, so these were ignored as well. There were 159 examples where ClustalW2 introduced one or more gaps involving more than one site. The significance of these examples varies in a continuous spectrum from many probably being of no significance through to the examples given in the Results, which were the two best. Logos and information content were calculated for the core of the sequence where base types were available for more than 50% of the binding sites. Information content for PWMs resulting from the gapped alignments and the standard alignments were calculated as described above.

## Results

### Investigation of TRANSFAC binding sites

**Figure 5:**
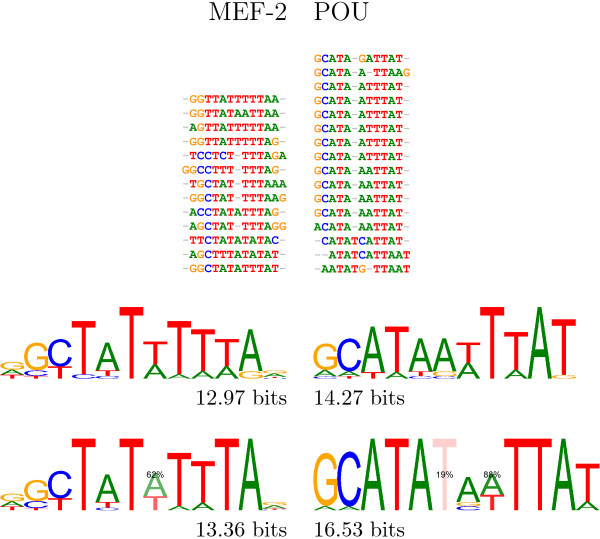
**Analysis of TRANSFAC binding sites**. Realignment of sequences used within TRANSFAC to define PWMs which incorporate optional gaps. **Left: **An alignment, a standard PWM and a gapped PWM for the monomer transcription factor MEF-2. Additional gaps improve the alignment right across the motif, especially the well conserved TA motif that is not apparent in the ungapped alignment. **Right: **An alignment, a standard PWM and a gapped PWM for the homodimer transcription factor POU. Additional gaps improve the alignment of the conserved ATA and TTA motifs. The realignments show a significant proportion of sites both with and without gaps. The upper logos show the original TRANSFAC motifs and their information content in bits (see Methods for the details of the calculation). The lower logos show the motifs after the addition of gaps, indicated by the percentage of sequences where a nucleotide is present.

An examination of the binding sites used to create the PWMs in the TRANSFAC database suggested that introducing gaps into the middle of binding sites could achieve a better alignment for the whole length of the motif. ClustalW2 [50] was used to identify the PWMs where the alignment could be improved by introducing gaps. Figure 5 shows two cases where gaps improve the alignment of well conserved *L*-mers on either side of the gaps. The improvement in the definition of the binding motif is also visible in the logos for the two transcription factors.

These are in addition to the 10 PWMs where gaps had already been introduced in the sequences to define the PWMs published in TRANSFAC. No recognition is made of this when the PWMs are used in motif scanning applications such as MATCH [51].

ClustalW2 also identified instances, such as V$AP4_Q6_01 where at least one of the binding sites within TRANSFAC had been misaligned with respect to the others.

### Motif finder comparison

We analysed six ChIP-seq data sets (see Table 1) with MEME, GLAM2, our novel gapped PWM method and a variant of our method in which the introduction of a gap is disabled. We also attempted to use the RISOTTO method but found it unsuitable for this task (for discussion see Additional file 1). For each data set we performed a cross-validation using held-out data (see Methods section for the details) and also ran the motif finders on the entire data sets to compare the motifs they found by hand. We present some of the results here, the full set of ROC curves and motifs are in Additional file 1.

**Table 1:** The data sets

TF	# Sequences	# bases	Publications
Sp1	296	207,325	Cawley et al. [60]
p53	524	480,238	Cawley et al. [60] and Wei et al. [13]
GABP	2,275	500,203	Valouev et al. [73]
NRSF	1,687	225,265	Johnson et al. [17]
STAT5a	737	94,250	Liao et al. [74]
STAT5b	144	19,379	Liao et al. [74]

The data sets we analysed with the motif finders.

We chose MEME as a representative of currently popular motif finders. Extensive comparisons have been made between motif finders for standard PWMs [23] so we decided to evaluate our method's performance relative to just one of the best performing and most popular. In our personal experience, MEME has outperformed other popular motif finders. GLAM2 and RISOTTO were selected because they appeared to be the best candidate competitors for the task of finding motifs of variable structure. Finally, we compared our method to itself but with gaps disabled.

The comparison was based on a five-fold cross-validation. We discriminated between held-out sequences from the data sets and a set of negative control sequences. It is notoriously difficult to choose representative negative sequence sets for evaluating motif search algorithms. We chose three different negative sets: a random selection of sequences from the genome, a randomly selected set of promoters, and shuffled versions of the held-out sequences. Other authors (for example, [20,23,25,52-55]) use both artificial or shuffled sequences and genomic sequences to evaluate their methods. We found some disagreement between which methods performed well when different negative data sets were used. In general, the randomly selected promoters were more difficult to discriminate against than the random genomic regions. One reason could be that promoter regions contain potential binding sites for the factor in question which for a variety of reasons are not represented in the ChIP data.

### Motif search results

#### Validation of Wei et al.'s refined p53 motif

**Figure 6:**
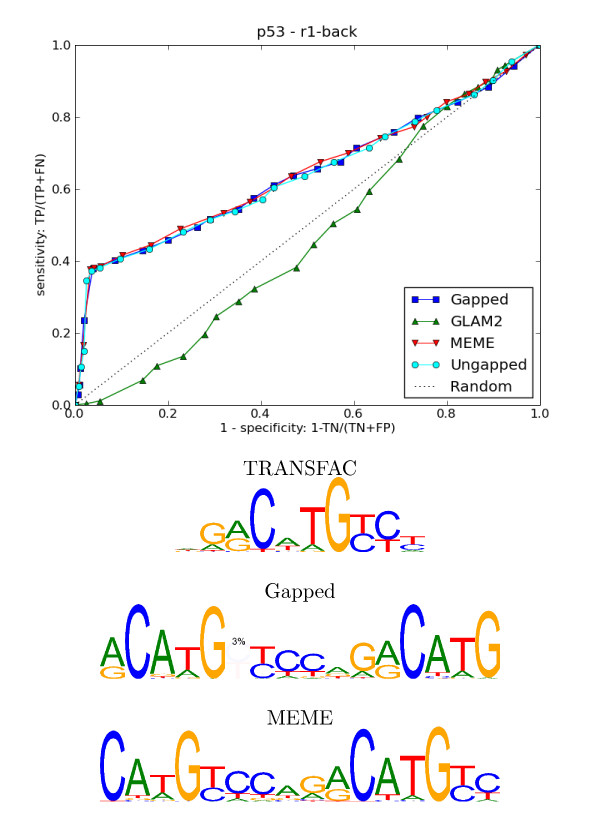
**p53 results**. **Top**: ROC curves for cross-validation on p53 data set using random genomic sequences as counter-examples. **Bottom**: A known TRANSFAC motif for p53 and the motifs our gapped method and MEME found. Using our method, 3% of the sites discovered had an optional spacer between the 2 half-sites. This is a close fit to Wei et al.'s analysis. They found 236 sites without a spacer and 27 that had a 1 base pair spacer.

Wei et al. [13] recover a refined version of the TRANSFAC p53 motif from their ChIP-seq experiments which consists of two p53 half-sites. Our method also recovers the refined motif (see Figure 6). One base pair of the variable inter-half-site spacing [5] is explicitly modelled by our motif. The ROC curves for MEME and our method are similar. This could be due to the low frequency of the extra base in the spacer (3%). GLAM2 discovered a very long vague motif in the data. Alu repeats can mutate into p53 binding sites [56]. This is the probable cause of the high level of unknown bases (~30%) in the p53 data set after repeat masking.

#### An improved Stat5 motif

**Figure 7:**
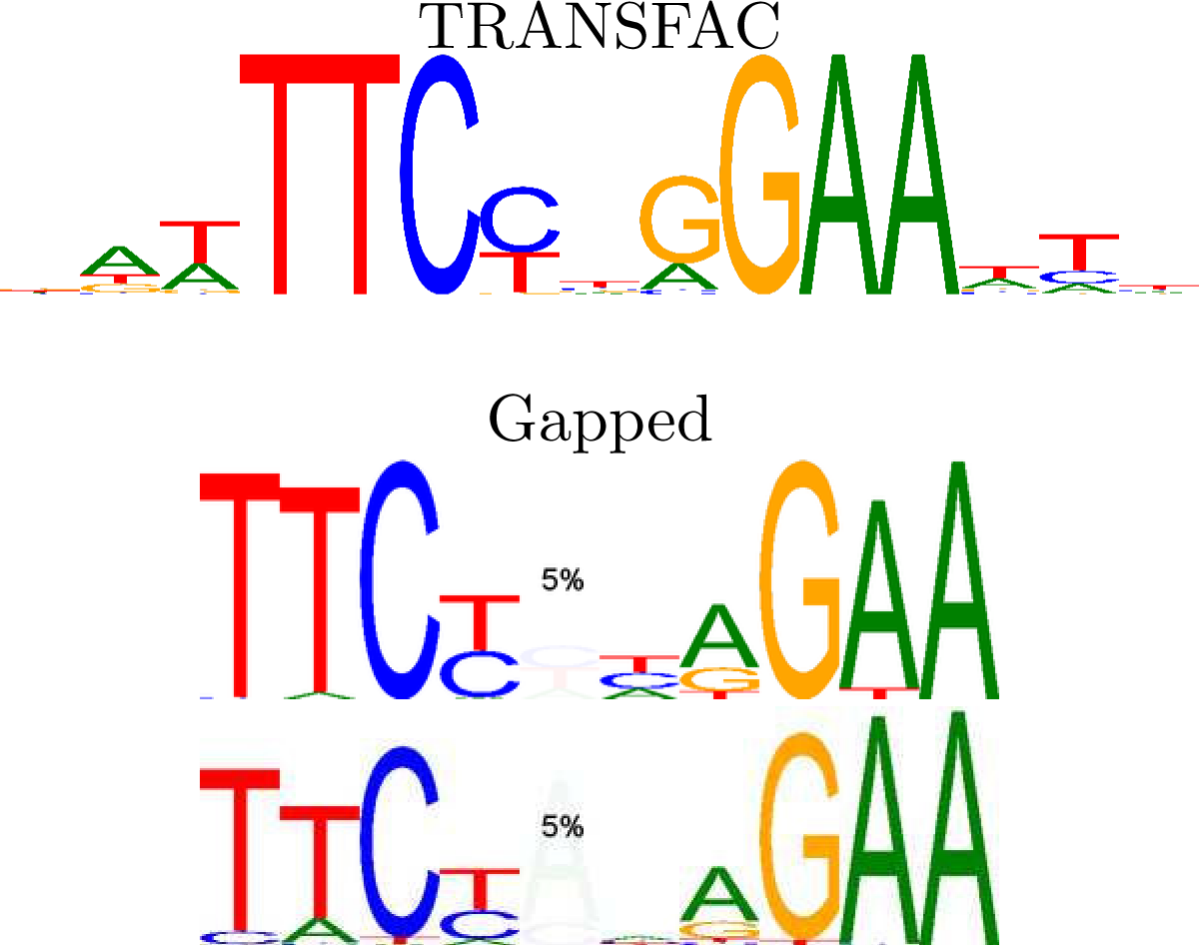
**Stat5 results**. A known TRANSFAC motif (M00459) for Stat5 and the motifs our method found in the Stat5a and Stat5b data sets.

Existing motifs for Stat5 in TRANSFAC do not incorporate any variable spacing between the 2 half-sites of the homodimer. Our method (and GLAM2) discovered that an extra base is present in 5% of the binding sites for Stat5a and Stat5b (see Figure 7). Furthermore, in the Stat5b case, the extra bases are almost exclusively adenines. These results agree with previously established hand-crafted HMM models of Stat binding [6]. Surprisingly, in cross-validation tests this extra base did not appear to improve the model. This could be due to multiple binding sites per sequence both with and without the extra base.

#### Recovery of GABP motif

All of the methods recovered a GABP motif extremely similar to the known TRANSFAC motif. Different methods performed differently in the cross-validation tests depending on the choice of negative control sequences. Neither our method nor GLAM2 found any evidence of significant variation in the spacing within the GABP motif.

#### GLAM2 discovers a variable motif for NRSF

**Figure 8:**
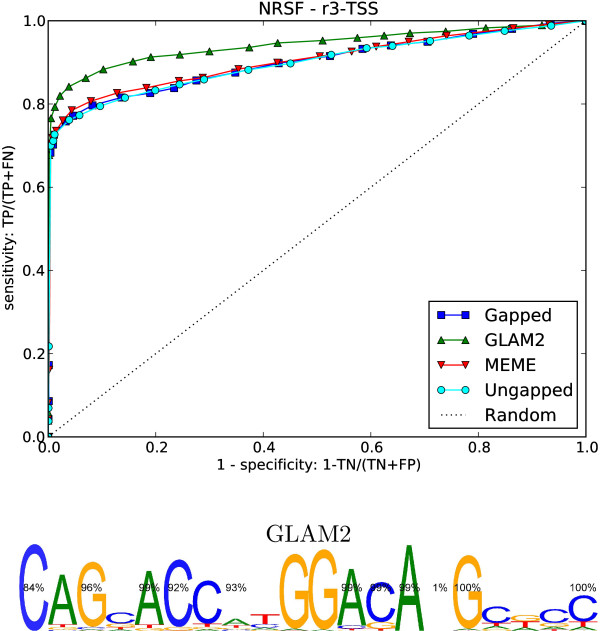
**NRSF results**. ROC curves for cross-validation on NRSF data set using random promoter sequences as counter-examples.

All of the methods recovered motifs very similar to the known NRSF binding motif. The GLAM2 motif makes many of the positions optional, albeit with probabilities close to 0 or 1 (see Figure 8). We might have dismissed this as an artifact of the GLAM2 algorithm if the cross-validation had not shown that this motif was clearly superior. NRSF is a zinc finger repressor that binds to a long (21 bp) DNA sequence motif known as the repressor element 1 (RE1) [57]. Bruce et al. [58] have established that variations in RE1 sites are associated with cell-type specific activity of NRSF. The variable structure that GLAM2 found may be associated with these effects.

#### A novel gapped Sp1 motif

**Figure 9:**
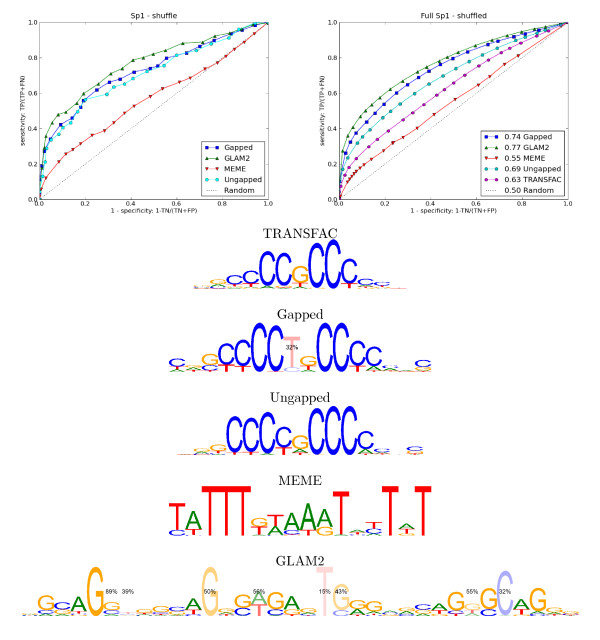
**Sp1 results**. **Top left**: ROC curves for cross-validation on Sp1 data set using shuffled versions of the held-out test sequences as counter-examples. **Top right**: ROC curves for the motifs found on the small data set when applied to a large Sp1 binding data set from TRANSFAC. The AUC statistics are given in the legend. **Bottom**: A known TRANSFAC motif for Sp1 (the reverse complement of M00196) and the motifs found by the methods we tested. In our model, 32% of the binding sites will have a T inserted after the fifth base. Note that modelling this optional base allows our method to avoid some ambiguity which is present in the Cs preceding the central G in the TRANSFAC motif.

Sp1 is a ubiquitous transcription factor that forms a part of the eukaryotic cellular transcriptional machinery and regulates many genes with GC-rich promoters [59]. Cawley et al. [60] note that the motif finding algorithm MDscan [22] recovers the known Sp1 binding motif from their data. Our method also recovers a similar motif but with an optional base that provides a more accurate model of the sites (see Figure 9). The information content of our motif is 13.14 bits compared to 9.64 bits for the TRANSFAC motif (see Methods section for details of calculation). Neither MEME nor GLAM2 recovered a motif similar to the known TRANSFAC motif. It is difficult to interpret the motif that GLAM2 found as the binding preferences of Sp1. It is possible that GLAM2 has found a long low-order feature in the data set which nevertheless has good predictive ability. Sp1 binding sites are common in the genome, especially in promoters [61]. Hence, we would expect to find Sp1 binding sites in many of the randomly selected negative examples. This is a probable cause of the poor cross-validation performance of the methods when random genomic regions or promoters were used as negative controls.

In order to assess whether the gapped motif was a better predictor of Sp1 binding than the known Sp1 motif, we took 125,063 Sp1 binding sequences comprising a total of 114,895,425 bases from a separate ChIP-chip data set in the TRANSFAC database [62] and tested how well the known TRANSFAC motif and the motifs discovered by the motif finders could distinguish these sequences from shuffled versions of the sequences, random genomic regions and randomly selected promoters. The results for the shuffled tests are shown in Figure 9 and the remainder are given in Additional file 1. The GLAM2 motif performed well in this test despite not resembling a motif for TFBSs. As mentioned, the GLAM2 motif might pick up a sequence signal beyond binding sites that characterises these regions: a repetitive GCAGG element, for example, is just discernible in the GLAM2 motif. Nevertheless, the gapped Sp1 motif found by our method out-performed the known TRANSFAC motif, the motif that MEME found and the motif found by the ungapped version of our algorithm when tested against shuffled sequences. When tested against promoters, none of the methods performed well, suggesting the promoters were not suitable as negative controls. The performance of the gapped motif against the random genomic regions was not as good as the TRANSFAC motif.

#### Overall results

**Figure 10:**
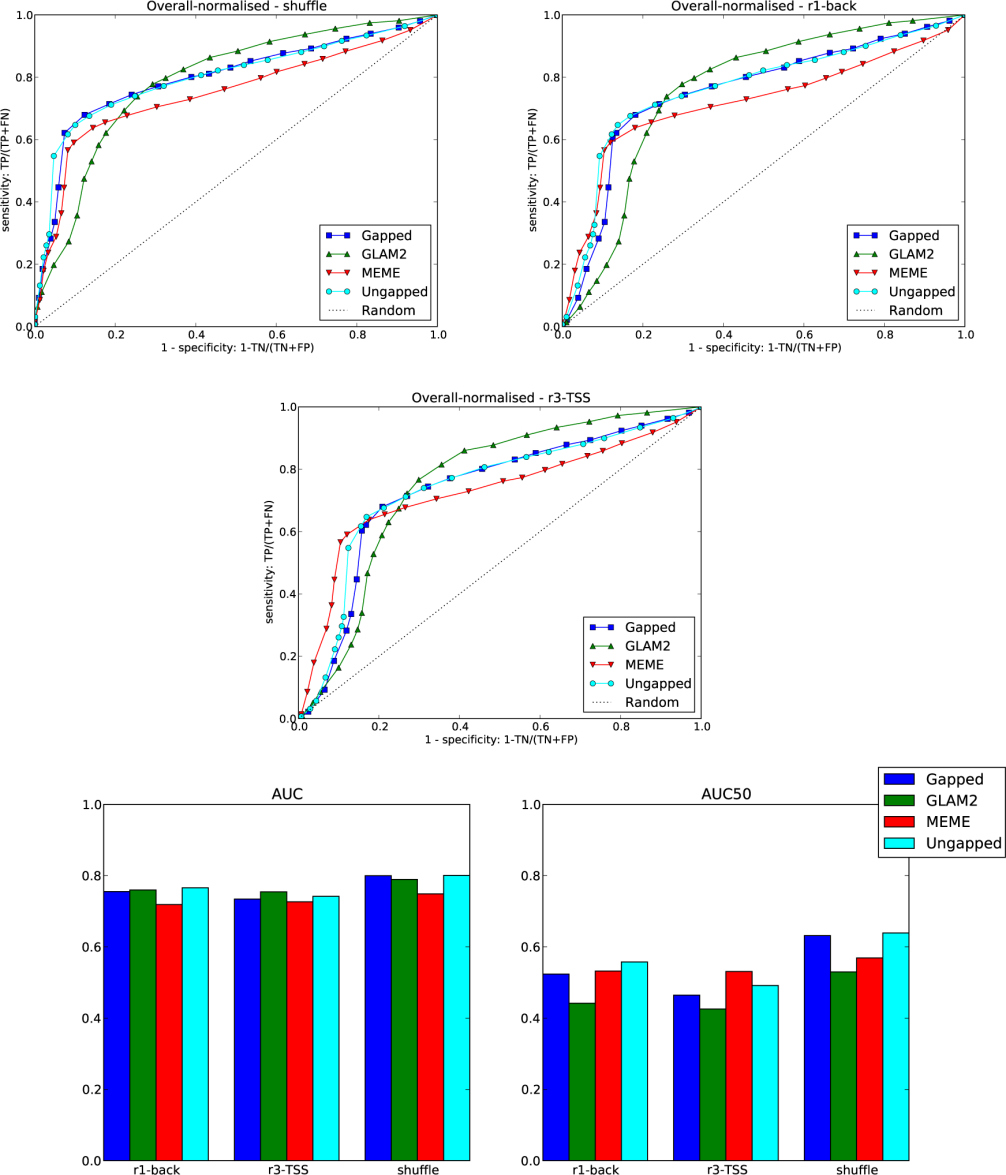
**Overall results**. **Top left**: ROC curves for cross-validation across all the data sets using shufflled versions of the held-out test sequences as counter-examples. **Top right**: ROC curves for cross-validation across all the data sets using randomly selected genomic sequences as counter-examples. **Middle**: ROC curves for cross-validation across all the data sets using randomly selected promoters as counter-examples. **Bottom**: AUC and AUC50 statistics for the methods.

Figure 10 shows ROC curves and AUC/AUC50 statistics that summarise the performance of the methods averaged over all the data sets. The methods have very similar AUC statistics although the choice of negative background sequences does affect which method perform best. The summaries shown here obscure significant differences between the methods on individual datasets. A complete set of AUC and AUC50 statistics is given in Additional file 1. Detailed examination of the individual AUC50 results, which give the performance of the methods at high specificity, shows the comparability of the methods.

## Discussion

### Our gapped method

Our tests demonstrate that our method performs comparably to MEME in cross-validation. In general, MEME performed better when tested against promoter sequences and our method was more successful against shuffled control sequences. However, the difference was not great in either case. Notably, our method retrieved a known Sp1 motif which MEME did not.

Compared to previous work on variable length motifs for p53 [5] and Stat5a [7], our method does not rely on prior knowledge of the structure of these sites. In general, we would not have prior knowledge about the structure of the binding sites. Methods that rely on it can only be used in the context of specific transcription factors.

We noticed little difference between the performance of our gapped method and the ungapped version of it. Despite this, we believe that the ability of the gapped method to recover the known variable Stat5 binding motifs and a novel gapped Sp1 motif is an important quality. We hope that discovery of novel gapped motifs of this type will lead to further structural studies to confirm or refute their validity.

### GLAM2

When GLAM2 found the correct motif it performed well. Even when it found long vague motifs with many gaps that seem biologically improbable (for p53 and Sp1), the cross-validated tests suggested it found some signal in the data. However, these motifs are difficult to interpret as models of TFBSs. Although the authors of GLAM2 mention motif finding as a possible application, they do not show an example in their paper. We believe our application of GLAM2 is the first to show its utility on realistically sized data sets in this context. We would also like to note (data not shown) that our experience with GLAM2 when no gaps or insertions are allowed shows it is a capable motif finder for standard PWMs.

Despite the overall ROC curves, we do not believe GLAM2 is inferior to our method or MEME in a predictive sense. When GLAM2 found a motif, it performed very well. It is disadvantaged in the overall results by its inability to recover a good p53 motif. GLAM2 was successful on those data sets which have a much shorter average sequence length. That is, those with a higher signal to noise ratio. It is perhaps best suited to data sets of this size.

The variable motif that GLAM2 found for NRSF bears further study, especially in light of the work done by Bruce et al. [58] relating variation in NRSF binding sites to lineage specific NRSF function.

### Negative controls

Our results for individual data sets varied according to which set of counter-examples was used for the ROC test. One reason we used three distinct sets of counter-examples was that there is no consensus in the literature about which classes of negative sequences to test against. Synthetically generated sequences are only an approximation to genomic sequences. They have the advantage that they control the expected number of false positives, however they can introduce a bias that favours one method over another [23]. On the other hand genomic sequences, either randomly selected or promoter regions, are similar to those sequences that the methods will be applied to in earnest. Unfortunately, they can frequently contain binding sites for the transcription factor of interest. This can also introduce biases or render the test insensitive.

Many factors influence transcription factor binding apart from the sequence. Perhaps a negative test set that took account of features associated with regulatory regions such as open chromatin regions and DNase hypersensitivity would be ideal. However, it would be difficult to construct such a test set without introducing other sources of bias as these features are dynamic and can vary between conditions.

### Structural basis for flexible spacing

**Figure 11:**
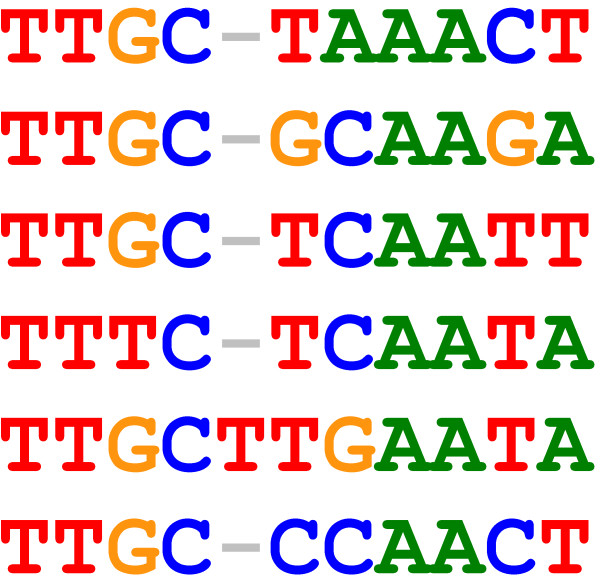
**C/EBP binding sites**. Binding sites for C/EBP.

Some classes of transcription factors are well known to allow flexible spacing in the recognised sequence. In particular, transcription factors forming multimers are able to accommodate variable distances between sequences recognised by each unit by readjusting the relative positions of the units. For example, leucine zippers consist of two *α *helices forming a fork. Recognition of variable spacing between the parts of the sequence motif recognised by each helix is possible by widening or narrowing the helices [63]. A possible example is shown in Figure 11 in an alignment of a selection of sequences of TRANSFAC motif M00912 bound by the mammalian transcription factor C/EBP, which forms a leucine zipper homodimer. The TRANSFAC consensus motif M00912 is approximated by TTNC{N}NNAANN, with curly brackets indicating an optional base.

In contrast, transcription factors of the basic HLH structure consist of four helices forming a rigid four helix bundle which prohibits any movement of its subunits and no variable spacing in the recognised sequence motif would be expected [63].

Presumably there is quite some difference in the binding energy between spacing variants, due to the need of rearranging contacts between multimer units. If such variants occur at all, most are expected to be relatively rare compared to the major spacing as seen in the C/EBP example.

An example where variants of spacing between units of a homodimer seem relatively common is seen in the alignment of sequences of TRANSFAC motif M00941 for MEF2 (see Figure 5). MEF2 belongs to the family of MADS-box proteins containing a common conserved 58-amino acid DNA-binding domain, the MADS-box [64]. An approximate consensus sequence is (C/t)TAT{T/a}(T/a)(A/t)TA(G/a) with a spacing of two base pairs between the (C/t)TAT and the (A/t)TA(G/a) palindromic binding motif of each unit of the homodimer. As our sequence examples show, it seems a reduced space of only one base pair between these units can be accommodated as well, as indicated by the curly bracket in the consensus sequence. It has to be said that structurally the dimer interface looks quite rigid, with two *β *sheets, one from each unit, aligned and two *α *helices, one from each unit stacked perpendicularly on top of the sheet, with the two *α *helices, which recognise the motif, below the sheet. Binding of MEF2 bends the DNA. Possibly, alternative spacings induce different bending angles in the DNA instead of inducing rearrangements in the dimer interface.

The p53 transcription factor is an anti-parallel *β *barrel with loop regions and a recognition *α *helix at the C-terminal side interacting with DNA [63]. It often seems to bind in tandem [5], that is with two adjacent recognition sites with approximate consensus CATGTC separated by variable spacing (see Figure 6). If two p53 proteins are bound at the same time they are close enough to make interaction likely.

Sp1 is an example of the family of C_2_H_2 _zinc finger transcription factors, with two cysteines and two histidines in coordination with a zinc atom providing structural stability. The loop region between the histidine and the cysteine residue binds the DNA. Zinc fingers are seen in tandem, proteins with several dozens of fingers exist [63]. Sp1 contains three zinc fingers binding consecutive base pair triplets with approximate consensus CCC, CGC, and CCC [65]. Zinc finger binding sites are known for their flexibility in base composition as well as in the length of the recognised motif, with three to five base pairs per finger. Our example shows that the middle zinc finger of Sp1 is possibly able to bind a three base pair motif CGC as well as one with four base pairs, CTGC. This flexibility would have to come from side chain rearrangements within the middle finger. Rearrangements between zinc fingers are unlikely.

Stat5a and Stat5b are members of the large family of Stat proteins. These more complex proteins form homodimers. A unit consists of an *α *helix bundle, a *β *barrel, an *α *helix connector region as well as an SH2 domain that forms the dimer interface. The *β *barrel and parts of the *α *helices interact with the DNA. It has been observed before that the Stat transcription factor is able to bind with different spacing between the motifs recognised by each unit of the dimer [66]. Presumably variations in spacing can be comparatively easily accommodated by rearrangements of the SH2 interfaces.

The POU region is part of several eukaryotic transcription factors. It consists of two DNA binding domains, a homeodomain and a POU specific domain. Both domains show no protein-protein contact and are linked by a 24 residue linker which is unordered and not visible in the crystal structure by Klemm et al. [67]. Due to this unordered connection between the domains, one might expect some flexibility in the spacing of the motifs recognised by each one: the POU specific domain binds the motif ATGC, whose reverse is seen in Figure 5, the homeodomain binds an A/T tetrad. There is a hint of flexible spacing between these motifs in Figure 5. The two domains would also have to bind in reverse order to the one in Klemm et al. [67] to explain this motif.

It has to be emphasised again that unless structures of the same DNA binding transcription factor under similar conditions but with different spacings between sub-motifs are available, the structural considerations above remain hypothetical. One of the aims of our study is to encourage further structural research of variable spacing.

### UniProbe

Berger and Bulyk have described a protocol [68] using universal protein binding microarrays to precisely determine the sequence binding preferences of transcription factors *in vitro*. Data for many transcription factors are available in their UniProbe database [69]. In light of this, motif search may become less relevant for those transcription factors assayed using this protocol. Nevertheless, we do not expect a comprehensive database of transcription factor binding preferences for all organisms to be available in the near future. Also the protocol has some limitations: it does not cater for transcription factors with long binding sites; neither can it accurately reproduce features of the *in vivo *system such as post-translational modifications and interactions with co-factors, both of which are known to affect binding preferences [70,71].

### Model limited to one gap

A natural extension to our model would be to allow the optional characters to span more than one base. For example, Pit1 is known to bind to sites that vary by two bases [4]. When we started work on this problem we investigated several models of this type. Our experience was that the numbers of parameters associated with these models quickly become too large. In other words, these models were too general and it was too easy to fit noise in the data, making inference difficult. Identifiability and interpretability were also issues: it was possible for different motifs under these models to produce almost identical distributions over words. These same issues were evident in our evaluation of GLAM2 for this problem. However, we believe extending the model slightly further in this direction is worthwhile. We have to leave a systematic evaluation of the performance of such variants for future work.

It is also possible to extend gapped PWMs to incorporate other generalisations of the PWM model such as position dependencies. However, we did not investigate position dependencies in this work.

## Conclusions

We have investigated the hypothesis that some transcription factors may exhibit a flexibility in their DNA interaction domains that allows for the recognition of variable length motifs. Whilst transcription factors that have variable length binding sites may not be the norm, we found evidence of them in several of the ChIP-seq data sets we tested. Furthermore we re-investigated known binding sites listed in TRANSFAC and found that allowing for gaps in binding sites can sharpen some existing motifs and improve their predictive power.

When we started this work, we were not aware of the previous hand-crafted variable length models for Stat5 and p53 binding sites. Our results have shown that our method is capable of finding and modelling these sites without any prior knowledge of their structure. The only other tool known to us that can find such motifs is GLAM2. GLAM2 has a more flexible model of TFBSs which allows it to find gapped motifs in some data sets. However, it appears to be too flexible to allow it to find plausible p53 and Sp1 motifs. In addition to recovering known motifs for Stat5 and p53 proteins, we discovered a variable length variation of the motif for Sp1 that models binding sites in ChIP-chip data more accurately.

Our gapped motif finder did not significantly outperform other methods in our cross-validation tests. Our results show its predictive ability is at least comparable to MEME. However, prediction is not the sole aim of motif search. Improving our models of transcription factor binding preferences is worthwhile in itself. In light of the evidence that regulatory function can depend on variations in the structure of binding sites [4], we believe further work in this relatively unexplored area should be performed.

When evaluated against other methods to search for motifs of variable structure, our method discovered previously known or interesting motifs in all the data sets we used. RISOTTO was not successful at all and GLAM2 achieved good results on four of the six data sets.

We have proposed a generalised model for transcription factor binding and provided an inference algorithm for efficient model fitting. We have evaluated our generalised binding model and found it to perform comparably to classical restricted motifs as found by MEME. Our new model makes an enhancement to PWMs and is complementary to other generalised models that take into account neighbourhood dependencies. We believe it will prove useful for incorporating information of variable binding sites into systems biology models of gene regulation.

Our motif search method and most other motif search methods only utilise sequence data. There is evidence [72] that other sources of data can help significantly in learning models of transcription factor binding preferences. Examples of such data sources include phylogenetic comparisons, epigenetic data, protein-protein interaction data, and binding site clustering analyses. The increasing availability of such data is likely to make methods that can utilise it more prevalent.

Variable length models may be able to better explain binding affinity fold-changes than nucleotide substitution models and this is an area for more research.

## Authors' contributions

SO conceived the study and participated in the design of the variable length motif models. KE prepared the data sets, performed the MEME, GLAM2 and RISOTTO comparisons and participated in the design of the variable length motif models. JR participated in the design of the variable length motif models, developed the variable length motif search algorithm, performed the GLAM2 cross-validation tests, built the evaluation framework and wrote the majority of the paper. ND analysed the TRANSFAC binding sites and prepared the data sets. LW participated in the design of the variable length motif models and wrote the structural discussion. All authors contributed to, read and approved the final manuscript. JR and KE would like to be considered as having contributed equally to this work.

## Software availability

The data sets used in the analysis and the source code for the algorithm are provided in Additional files 2 and 3.

## Supplementary Material

Additional file 1 **Supplementary materials**. This document contains a full set of results, the technical details of the evaluations and a discussion of the motif finder RISOTTO. Click here for file

Additional file 2 **Data sets**. A bzipped archive of the processed data sets used in the evaluations. Click here for file

Additional file 3 **Application source code**. The source code of the implementation of our method. Click here for file

## References

[B1] Loh YH Wu Q Chew JL Vega VB Zhang W Chen X Bourque G George J Leong B Liu J Wong KY Sung KW Lee CW Zhao XD Chiu KP Lipovich L Kuznetsov VA Robson P Stanton LW Wei CL Ruan Y Lim B Ng HH The Oct4 and Nanog transcription network regulates pluripotency in mouse embryonic stem cells Nat Genet 2006 38 4 431 40 10.1038/ng1760 16518401

[B2] Tanay A Extensive low-affinity transcriptional interactions in the yeast genome Genome Res 2006 16 8 962 72 10.1101/gr.5113606 16809671 PMC1524868

[B3] Foat BC Morozov AV Bussemaker HJ Statistical mechanical modeling of genome-wide transcription factor occupancy data by MatrixREDUCE Bioinformatics 2006 22 14 e141 9 10.1093/bioinformatics/btl223 16873464

[B4] Scully KM Jacobson EM Jepsen K Lunyak V Viadiu H Carrière C Rose DW Hooshmand F Aggarwal AK Rosenfeld MG Allosteric effects of Pit-1 DNA sites on long-term repression in cell type specification Science 2000 290 5494 1127 1131 10.1126/science.290.5494.1127 11073444

[B5] Riley T Sontag E Chen P Levine A Transcriptional control of human p53-regulated genes Nat Rev Mol Cell Biol 2008 9 5 402 412 10.1038/nrm2395 18431400

[B6] Soldaini E John S Moro S Bollenbacher J Schindler U Leonard WJ DNA binding site selection of dimeric and tetrameric Stat5 proteins reveals a large repertoire of divergent tetrameric Stat5a binding sites Mol Cell Biol 2000 20 389 401 10.1128/MCB.20.1.389-401.2000 10594041 PMC85094

[B7] Ehret GB Reichenbach P Schindler U Horvath CM Fritz S Nabholz M Bucher P DNA binding specificity of different STAT proteins. Comparison of in vitro specificity with natural target sites J Biol Chem 2001 276 9 6675 6688 10.1074/jbc.M001748200 11053426

[B8] Tan T Chu G p53 Binds and activates the xeroderma pigmentosum DDB2 gene in humans but not mice Mol Cell Biol 2002 22 10 3247 3254 10.1128/MCB.22.10.3247-3254.2002 11971958 PMC133779

[B9] Tuerk C Gold L Systematic evolution of ligands by exponential enrichment: RNA ligands to bacteriophage T4 DNA polymerase Science 1990 249 4968 505 510 10.1126/science.2200121 2200121

[B10] Matys V Kel-Margoulis OV Fricke E Liebich I Land S Barre-Dirrie A Reuter I Chekmenev D Krull M Hornischer K Voss N Stegmaier P Lewicki-Potapov B Saxel H Kel AE Wingender E TRANSFAC and its module TRANSCompel: transcriptional gene regulation in eukaryotes Nucleic Acids Res 2006 34 Database D108 D110 10.1093/nar/gkj143 16381825 PMC1347505

[B11] Sandelin A Alkema W Engstrom P Wasserman WW Lenhard B JASPAR: an open-access database for eukaryotic transcription factor binding profiles Nucleic Acids Res 2004 32 D91 D94 10.1093/nar/gkh012 14681366 PMC308747

[B12] Lee TI Jenner RG Boyer LA Guenther MG Levine SS Kumar RM Chevalier B Johnstone SE Cole MF ichi Isono K Koseki H Fuchikami T Abe K Murray HL Zucker JP Yuan B Bell GW Herbolsheimer E Hannett NM Sun K Odom DT Otte AP Volkert TL Bartel DP Melton DA Gifford DK Jaenisch R Young RA Control of developmental regulators by Polycomb in human embryonic stem cells Cell 2006 125 2 301 313 10.1016/j.cell.2006.02.043 16630818 PMC3773330

[B13] Wei CL Wu Q Vega VB Chiu KP Ng P Zhang T Shahab A Yong HC Fu Y Weng Z Liu J Zhao XD Chew JL Lee YL Kuznetsov VA Sung WK Miller LD Lim B Liu ET Yu Q Ng HH Ruan Y A global map of p53 transcription-factor binding sites in the human genome Cell 2006 124 207 219 10.1016/j.cell.2005.10.043 16413492

[B14] Kim TH Abdullaev ZK Smith AD Ching KA Loukinov DI Green RD Zhang MQ Lobanenkov VV Ren B Analysis of the vertebrate insulator protein CTCF-binding sites in the human genome Cell 2007 128 6 1231 1245 10.1016/j.cell.2006.12.048 17382889 PMC2572726

[B15] Boyer LA Lee TI Cole MF Johnstone SE Levine SS Zucker JP Guenther MG Kumar RM Murray HL Jenner RG Gifford DK Melton DA Jaenisch R Young RA Core transcriptional regulatory circuitry in human embryonic stem cells Cell 2005 122 6 947 956 10.1016/j.cell.2005.08.020 16153702 PMC3006442

[B16] Robertson G Hirst M Bainbridge M Bilenky M Zhao Y Zeng T Euskirchen G Bernier B Varhol R Delaney A Thiessen N Griffith OL He A Marra M Snyder M Jones S Genome-wide profiles of STAT1 DNA association using chromatin immunoprecipitation and massively parallel sequencing Nat Methods 2007 4 8 651 657 10.1038/nmeth1068 17558387

[B17] Johnson DS Mortazavi A Myers RM Wold B Genome-wide mapping of in vivo protein-DNA interactions Science 2007 316 5830 1497 1502 10.1126/science.1141319 17540862

[B18] Harbison CT Gordon BD Lee TI Rinaldi NJ Macisaac KD Danford TW Hannett NM Tagne JB Reynolds DB Yoo J Jennings EG Zeitlinger J Pokholok DK Kellis M Rolfe AP Takusagawa KT Lander ES Gifford DK Fraenkel E Young RA Transcriptional regulatory code of a eukaryotic genome Nature 2004 431 7004 99 104 10.1038/nature02800 15343339 PMC3006441

[B19] Bailey TL Williams N Misleh C Li WW MEME: discovering and analyzing DNA and protein sequence motifs Nucleic Acids Res 2006 34 Web Server W369 W373 10.1093/nar/gkl198 16845028 PMC1538909

[B20] Down TA Hubbard TJP NestedMICA: sensitive inference of over-represented motifs in nucleic acid sequence Nucleic Acids Res 2005 33 5 1445 1453 10.1093/nar/gki282 15760844 PMC1064142

[B21] Roth FP Hughes JD Estep PW Church GM Finding DNA regulatory motifs within unaligned noncoding sequences clustered by whole-genome mRNA quantitation Nat Biotechnol 1998 16 10 939 945 10.1038/nbt1098-939 9788350

[B22] Liu XS Brutlag DL Liu JS An algorithm for finding protein-DNA binding sites with applications to chromatin-immunoprecipitation microarray experiments Nat Biotechnol 2002 20 8 835 839 12101404 10.1038/nbt717

[B23] Tompa M Li N Bailey TL Church GM De Moor B Eskin E Favorov AV Frith MC Fu Y Kent WJ Makeev VJ Mironov AA Noble WS Pavesi G Pesole G Regnier M Simonis N Sinha S Thijs G van Helden J Vandenbogaert M Weng Z Workman C Ye C Zhu Z Assessing computational tools for the discovery of transcription factor binding sites Nat Biotechnol 2005 23 137 44 10.1038/nbt1053 15637633

[B24] Sandve GK Abul O Walseng V Drabløs F Improved benchmarks for computational motif discovery BMC Bioinformatics 2007 8 193 10.1186/1471-2105-8-193 17559676 PMC1903367

[B25] Eden E Lipson D Yogev S Yakhini Z Discovering motifs in ranked lists of DNA sequences PLoS Comput Biol 2007 3 3 e39 10.1371/journal.pcbi.0030039 17381235 PMC1829477

[B26] Redhead E Bailey TL Discriminative motif discovery in DNA and protein sequences using the DEME algorithm BMC Bioinformatics 2007 8 385 10.1186/1471-2105-8-385 17937785 PMC2194741

[B27] Morozov AV Siggia ED Connecting protein structure with predictions of regulatory sites Proc Natl Acad Sci USA 2007 104 17 7068 73 10.1073/pnas.0701356104 17438293 PMC1855371

[B28] Day WH McMorris FR Critical comparison of consensus methods for molecular sequences Nucleic Acids Res 1992 20 5 1093 1099 10.1093/nar/20.5.1093 1549472 PMC312096

[B29] Waterman Introduction to Computational Biology 1995 chap. 2 Chapman and Hall, London

[B30] Sharon E Lubliner S Segal E A feature-based approach to modeling protein-DNA interactions PLoS Comput Biol 2008 4 8 e1000154 10.1371/journal.pcbi.1000154 18725950 PMC2516605

[B31] van Helden J Rios AF Collado-Vides J Discovering regulatory elements in non-coding sequences by analysis of spaced dyads Nucleic Acids Res 2000 28 8 1808 1818 10.1093/nar/28.8.1808 10734201 PMC102821

[B32] Carvalho AM Freitas AT Oliveira AL Sagot MF An efficient algorithm for the identification of structured motifs in DNA promoter sequences IEEE/ACM Trans Comput Biol Bioinform 2006 3 2 126 140 10.1109/TCBB.2006.16 17048399

[B33] Brazma A Jonassen I Eidhammer I Gilbert D Approaches to the automatic discovery of patterns in biosequences J Comput Biol 1998 5 2 279 305 10.1089/cmb.1998.5.279 9672833

[B34] Frith MC Saunders NFW Kobe B Bailey TL Discovering sequence motifs with arbitrary insertions and deletions PLoS Comput Biol 2008 4 4 e1000071 10.1371/journal.pcbi.1000071 18437229 PMC2323616

[B35] Riley T Yu X Sontag E Levine A The p53HMM algorithm: using profile hidden markov models to detect p53-responsive genes BMC Bioinformatics 2009 10 111 10.1186/1471-2105-10-111 19379484 PMC2685388

[B36] Badis G Berger MF Philippakis AA Talukder S Gehrke AR Jaeger SA Chan ET Metzler G Vedenko A Chen X Kuznetsov H Wang CF Coburn D Newburger DE Morris Q Hughes TR Bulyk ML Diversity and Complexity in DNA Recognition by Transcription Factors Science 2009 324 1720 1723 10.1126/science.1162327 19443739 PMC2905877

[B37] Stormo GD DNA binding sites: representation and discovery Bioinformatics 2000 16 16 23 10.1093/bioinformatics/16.1.16 10812473

[B38] Rabiner LR A tutorial on hidden Markov models and selected applications in speech recognition Proceedings of the IEEE 1989 77 2 257 286 10.1109/5.18626

[B39] Casella G George EI Explaining the Gibbs Sampler The American Statistician 1992 46 3 167 174 10.2307/2685208

[B40] Lawrence CE Altschul SF Boguski MS Liu JS Neuwald AF Wootton JC Detecting subtle sequence signals: a Gibbs sampling strategy for multiple alignment Science 1993 262 5131 208 214 10.1126/science.8211139 8211139

[B41] Hughes JD Estep PW Tavazoie S Church GM Computational identification of cis-regulatory elements associated with groups of functionally related genes in Saccharomyces cerevisiae J Mol Biol 2000 296 5 1205 1214 10.1006/jmbi.2000.3519 10698627

[B42] Workman CT Stormo GD ANN-Spec: a method for discovering transcription factor binding sites with improved specificity Pac Symp Biocomput 2000 467 478 10902194 10.1142/9789814447331_0044

[B43] Thijs G Marchal K Lescot M Rombauts S Moor BD Rouzé P Moreau Y A Gibbs sampling method to detect overrepresented motifs in the upstream regions of coexpressed genes J Comput Biol 2002 9 2 447 464 10.1089/10665270252935566 12015892

[B44] Frith MC Hansen U Spouge JL Weng Z Finding functional sequence elements by multiple local alignment Nucleic Acids Res 2004 32 189 200 10.1093/nar/gkh169 14704356 PMC373279

[B45] Favorov AV Gelfand MS Gerasimova AV Ravcheev DA Mironov AA Makeev VJ A Gibbs sampler for identification of symmetrically structured, spaced DNA motifs with improved estimation of the signal length Bioinformatics 2005 21 10 2240 2245 10.1093/bioinformatics/bti336 15728117

[B46] Chen X Guo L Fan Z Jiang T W-AlignACE: an improved Gibbs sampling algorithm based on more accurate position weight matrices learned from sequence and gene expression/ChIP-chip data Bioinformatics 2008 24 9 1121 1128 10.1093/bioinformatics/btn088 18325926

[B47] Bailey TL Elkan C Fitting a mixture model by expectation maximization to discover motifs in biopolymers Proceedings of the Second International Conference on Intelligent Systems for Molecular Biology 1994 AAAI Press 28 36 7584402

[B48] Gusfield D Algorithms on strings, trees, and sequences: computer science and computational biology 2007 Cambridge Univ. Press

[B49] Gribskov M Veretnik S Identification of sequence pattern with profile analysis Methods Enzymol 1996 266 198 212 full_text 8743686 10.1016/s0076-6879(96)66015-7

[B50] Larkin MA Blackshields G Brown NP Chenna R McGettigan PA McWilliam H Valentin F Wallace IM Wilm A Lopez R Thompson JD Gibson TJ Higgins DG Clustal W and Clustal X version 2.0 Bioinformatics 2007 23 21 2947 2948 10.1093/bioinformatics/btm404 17846036

[B51] Kel AE Gössling E Reuter I Cheremushkin E Kel-Margoulis OV Wingender E MATCH: A tool for searching transcription factor binding sites in DNA sequences Nucleic Acids Res 2003 31 13 3576 3579 10.1093/nar/gkg585 12824369 PMC169193

[B52] Shida K GibbsST: a Gibbs sampling method for motif discovery with enhanced resistance to local optima BMC Bioinformatics 2006 7 486 10.1186/1471-2105-7-486 17083740 PMC1647290

[B53] Wang T Stormo GD Combining phylogenetic data with co-regulated genes to identify regulatory motifs Bioinformatics 2003 19 18 2369 2380 10.1093/bioinformatics/btg329 14668220

[B54] Siddharthan R Siggia ED van Nimwegen E PhyloGibbs: a Gibbs sampling motif finder that incorporates phylogeny PLoS Comput Biol 2005 1 7 e67 10.1371/journal.pcbi.0010067 16477324 PMC1309704

[B55] Sinha S Blanchette M Tompa M PhyME: a probabilistic algorithm for finding motifs in sets of orthologous sequences BMC Bioinformatics 2004 5 170 10.1186/1471-2105-5-170 15511292 PMC534098

[B56] Zemojtel T Kielbasa SM Arndt PF Chung HR Vingron M Methylation and deamination of CpGs generate p53-binding sites on a genomic scale Trends Genet 2009 25 2 63 66 10.1016/j.tig.2008.11.005 19101055

[B57] Chong JA Tapia-Ramírez J Kim S Toledo-Aral JJ Zheng Y Boutros MC Altshuller YM Frohman MA Kraner SD Mandel G REST: a mammalian silencer protein that restricts sodium channel gene expression to neurons Cell 1995 80 6 949 957 10.1016/0092-8674(95)90298-8 7697725

[B58] Bruce AW López-Contreras AJ Flicek P Down TA Dhami P Dillon SC Koch CM Langford CF Dunham I Andrews RM Vetrie D Functional diversity for REST (NRSF) is defined by in vivo binding affinity hierarchies at the DNA sequence level Genome Res 2009 19 6 994 1005 10.1101/gr.089086.108 19401398 PMC2694481

[B59] Kaczynski J Cook T Urrutia R Sp1- and Krüppel-like transcription factors Genome Biol 2003 4 2 206 10.1186/gb-2003-4-2-206 12620113 PMC151296

[B60] Cawley S Bekiranov S Ng HH Kapranov P Sekinger EA Kampa D Piccolboni A Sementchenko V Cheng J Williams AJ Wheeler R Wong B Drenkow J Yamanaka M Patel S Brubaker S Tammana H Helt G Struhl K Gingeras TR Unbiased mapping of transcription factor binding sites along human chromosomes 21 and 22 points to widespread regulation of noncoding RNAs Cell 2004 116 4 499 509 10.1016/S0092-8674(04)00127-8 14980218

[B61] Wierstra I Sp1: Emerging roles-Beyond constitutive activation of TATA-less housekeeping genes Biochemical and Biophysical Research Communications 2008 372 1 13 10.1016/j.bbrc.2008.03.074 18364237

[B62] TRANSFAC New ChIP-on-chip data Rel121 2008

[B63] Brändén C Tooze J Introduction to protein structure 1991 Garland Publishing, New York

[B64] Santelli E Richmond TJ Crystal structure of MEF2A core bound to DNA at 1.5 A resolution J Mol Biol 2000 297 2 437 449 10.1006/jmbi.2000.3568 10715212

[B65] Oka S Shiraishi Y Yoshida T Ohkubo T Sugiura Y Kobayashi Y NMR structure of transcription factor Sp1 DNA binding domain Biochemistry 2004 43 51 16027 16035 10.1021/bi048438p 15609997

[B66] Seidel HM Milocco LH Lamb P Darnell JE Stein RB Rosen J Spacing of palindromic half sites as a determinant of selective STAT (signal transducers and activators of transcription) DNA binding and transcriptional activity Proc Natl Acad Sci USA 1995 92 7 3041 3045 10.1073/pnas.92.7.3041 7708771 PMC42355

[B67] Klemm JD Rould MA Aurora R Herr W Pabo CO Crystal structure of the Oct-1 POU domain bound to an octamer site: DNA recognition with tethered DNA-binding modules Cell 1994 77 21 32 10.1016/0092-8674(94)90231-3 8156594

[B68] Berger MF Bulyk ML Universal protein-binding microarrays for the comprehensive characterization of the DNA-binding specificities of transcription factors Nat Protoc 2009 4 3 393 411 10.1038/nprot.2008.195 19265799 PMC2908410

[B69] Newburger DE Bulyk ML UniPROBE: an online database of protein binding microarray data on protein-DNA interactions Nucleic Acids Res 2009 37 Database D77 D82 10.1093/nar/gkn660 18842628 PMC2686578

[B70] Wilson DS Desplan C Structural basis of Hox specificity Nat Struct Biol 1999 6 4 297 300 10.1038/7524 10201389

[B71] Joshi R Passner JM Rohs R Jain R Sosinsky A Crickmore MA Jacob V Aggarwal AK Honig B Mann RS Functional specificity of a Hox protein mediated by the recognition of minor groove structure Cell 2007 131 3 530 543 10.1016/j.cell.2007.09.024 17981120 PMC2709780

[B72] Hannenhalli S Eukaryotic transcription factor binding sites-modeling and integrative search methods Bioinformatics 2008 24 11 1325 1331 10.1093/bioinformatics/btn198 18426806

[B73] Valouev A Johnson DS Sundquist A Medina C Anton E Batzoglou S Myers RM Sidow A Genome-wide analysis of transcription factor binding sites based on ChIP-Seq data Nat Methods 2008 5 9 829 834 10.1038/nmeth.1246 19160518 PMC2917543

[B74] Liao W Schones DE Oh J Cui Y Cui K Roh TY Zhao K Leonard WJ Priming for T helper type 2 differentiation by interleukin 2-mediated induction of interleukin 4 receptor alpha-chain expression Nat Immunol 2008 9 11 1288 1296 10.1038/ni.1656 18820682 PMC2762127

