# Supplementary material for: Variable structure motifs for transcription factor binding sites
Source: BMC Genomics. 2010 Jan 14;11:30. doi: 10.1186/1471-2164-11-30 (PMC2824720; doi:10.1186/1471-2164-11-30)
Supplement: Additional file 1 — Supplementary materials. This document contains a full set of results, the technical details of the evaluations and a discussion of the motif finder RISOTTO. [file 1471-2164-11-30-S1.PDF]

Evaluation

MEME

MEME [1] version 3.5.7 was used with options "-dna -mod zoops -revcomp -text -w 16 -maxsize 30000000 -maxiter 150 -nmotifs 5". We have found that using "zoops" almost always gives a better balance between sequences and resulting ROC curves than using "anr". We have used the motif reported by MEME as giving the most sites, as this rule has been found to avoid degenerate motifs of nearly all Ts which MEME sometimes gives as most statistically significant. In fact, for most of these data sets this rule is identical to using the motif MEME reports as most statistically significant (there is a small exception for Sp1 where all motifs given by MEME give poor ROC curves).

GLAM2

GLAM2 allows complete generality of insertions and gaps throughout the length of the motif. We used the parameters to turn off insertions because otherwise the method reports very long biologically implausible runs of insertions. This still gives a huge parameter search space and it is not surprising that the method has difficulty in finding the global optimum. Even when the *-n* parameter (number of allowed iterations without improvement in the objective function) was increased to five hundred thousand from its default value of ten thousand, the number and position of gaps as well as the length of the motifs were sensitive to the seed given to the algorithm. We tried GLAM2 on all the data sets examined in this paper for eight replications using parameters "-2 -s S -r 1 -I 0.1 -J 99999.0 -n 500000" where the seed S took the values 1 to 8. For each data set we report the motif with the highest significance score.

Test data sets

The data for p53 and Sp1 were extracted from TRANSFAC professional 11.4 and the flanking bases added by TRANSFAC were removed. The data sets for GABP, NRSF, Stat5a and Stat5b were processed to extract the binding site sequences using the cisGenome software suite v1.0 [2]. In every case both sequences and controls were used. Binding region boundary refinement was used and then the region extended on each side by 30bp. For NRSF peaks were selected if there were more than 8 reads in a rolling 100 bp sequence window compared to the control which detected 1775 peaks. For the GABP data, peaks were selected if there were more than 18 reads in a rolling 100 bp sequence window compared to the control. This higher figure was selected to remove visually noisy peaks and 10767 peaks were detected. Cutoffs of 30 and 20 reads were used for the Stat5a and Stat5b data respectively yielding 814 and 154 peaks. RepeatMasker was used on all the test data sets to mask repetitive elements using the genomic context for each sequence. We provide the sequences as part of the Supplementary Materials. These files give data for sequences and genomic coordinates. The results in the paper are based on the masked data, but the unmasked data is given for completeness. The sequences are given in FASTA format and notes about the files for genomic coordinates (including assembly versions) are given within the files.

ROC curves, AUC and AUC50 statistics

We evaluated each method on each of the datasets, using three different background data sets as negative examples. These evaluations are presented as ROC curves and AUC and AUC50 statistics. Given a method, M, a set of positive examples, X^+, and a set of negative examples, X^-, the details of the evaluations are as follows.

Let $M(x)$ be the score the method gives example x, then we define the positive and negative scores to be

$$S^+ = \{M(x) : x \in X^+\}, \qquad S^- = \{M(x) : x \in X^-\}$$

For a given threshold, t, we define the number of true positives, false positives, true negatives and false negatives as

$$\mathrm{TP} = |\{s : s \in S^+, s \geq t\}|, \qquad \mathrm{FP} = |\{s : s \in S^-, s \geq t\}|, \qquad \mathrm{TN} = |\{s : s \in S^-, s < t\}|, \qquad \mathrm{FN} = |\{s : s \in S^+, s < t\}|$$

The specificity and sensitivity are defined as

$$\text{sensitivity} = \frac{\mathrm{TP}}{\mathrm{TP} + \mathrm{FN}}, \qquad \text{specificity} = \frac{\mathrm{TN}}{\mathrm{TN} + \mathrm{FP}}$$

The ROC curve is a plot of 1-specificity as the abscissa against sensitivity as the ordinate for various thresholds. The AUC is the area under the ROC curve and can be interpreted as the probability that a randomly chosen positive example will score more highly than a randomly chosen negative example under the given method. The AUC is a common metric used to assess ROC curves.

Oftentimes a user will apply a method to many examples and can only afford to perform follow-up investigations on a few of these. These users will be interested in the performance of the method at a high specificity, that is when the false positive rate is low. The AUC50 [3] is a statistic to measure this ability. We define $X^-_{M,50}$ as the 50

highest scoring negative examples under the method. Using the original set of positive examples, X^+, and the new negative examples, $X^-_{M,50}$, we generate another ROC curve (called the ROC50 curve). The area under this curve is the AUC50 statistic. The AUC50 statistic can be interpreted as the probability that a positive example will be correctly classified over a randomly selected high-scoring negative example.

Cross-validated ROC curves

We give the results for each data set individually. We show ROC curves using three different negative sets of sequences:

- *shuffle* : Shuffled versions of the positive sequences
- *r1-back* : Random genomic regions
- *r3-TSS* : Randomly selected promoters

The top 3 figures are ROC curves for the different negative sets of sequences. Following the ROC curves we show precision-recall curves. The bottom figures show the AUC and AUC50 statistics. We show the logos of the motifs discovered by each method when run on the whole data set.

p53

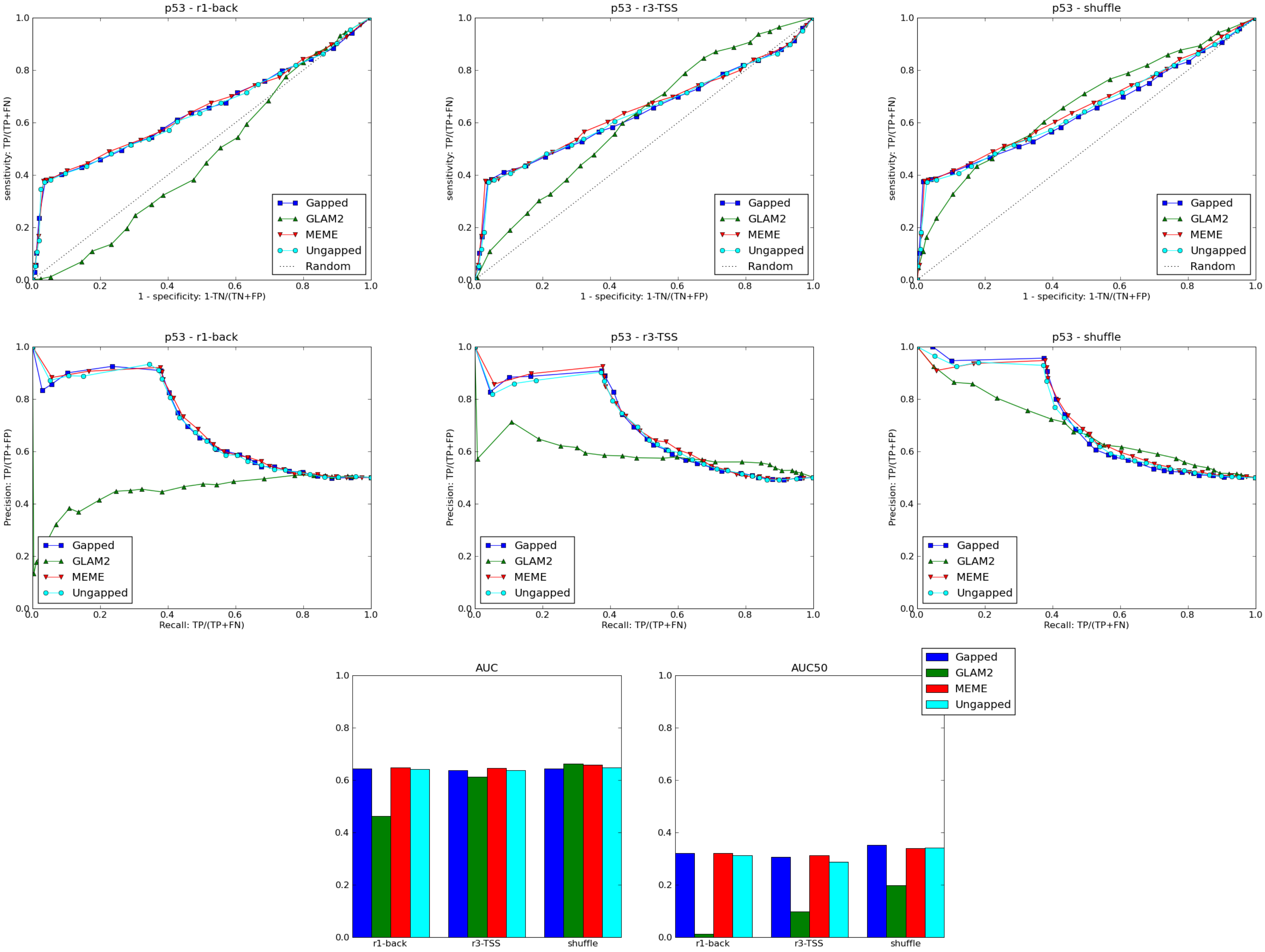

p53 - r1-back
p53 - r3-TSS
p53 - shuffle
sensitivity: TP/(TP+FN)
1 - specificity: 1-TN/(TN+FP)
Precision: TP/(TP+FP)
Recall: TP/(TP+FN)
Gapped
GLAM2
MEME
Ungapped
Random
AUC
AUC50
r1-back
r3-TSS
shuffle

Gapped

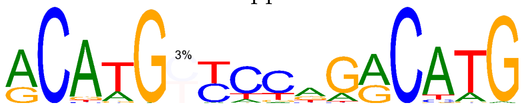

Ungapped

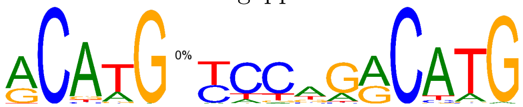

MEME

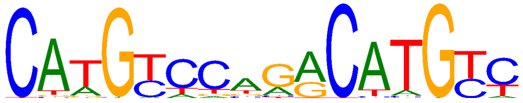

GLAM2

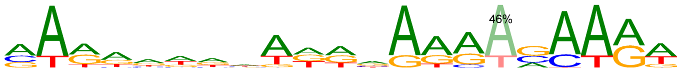

Sp1

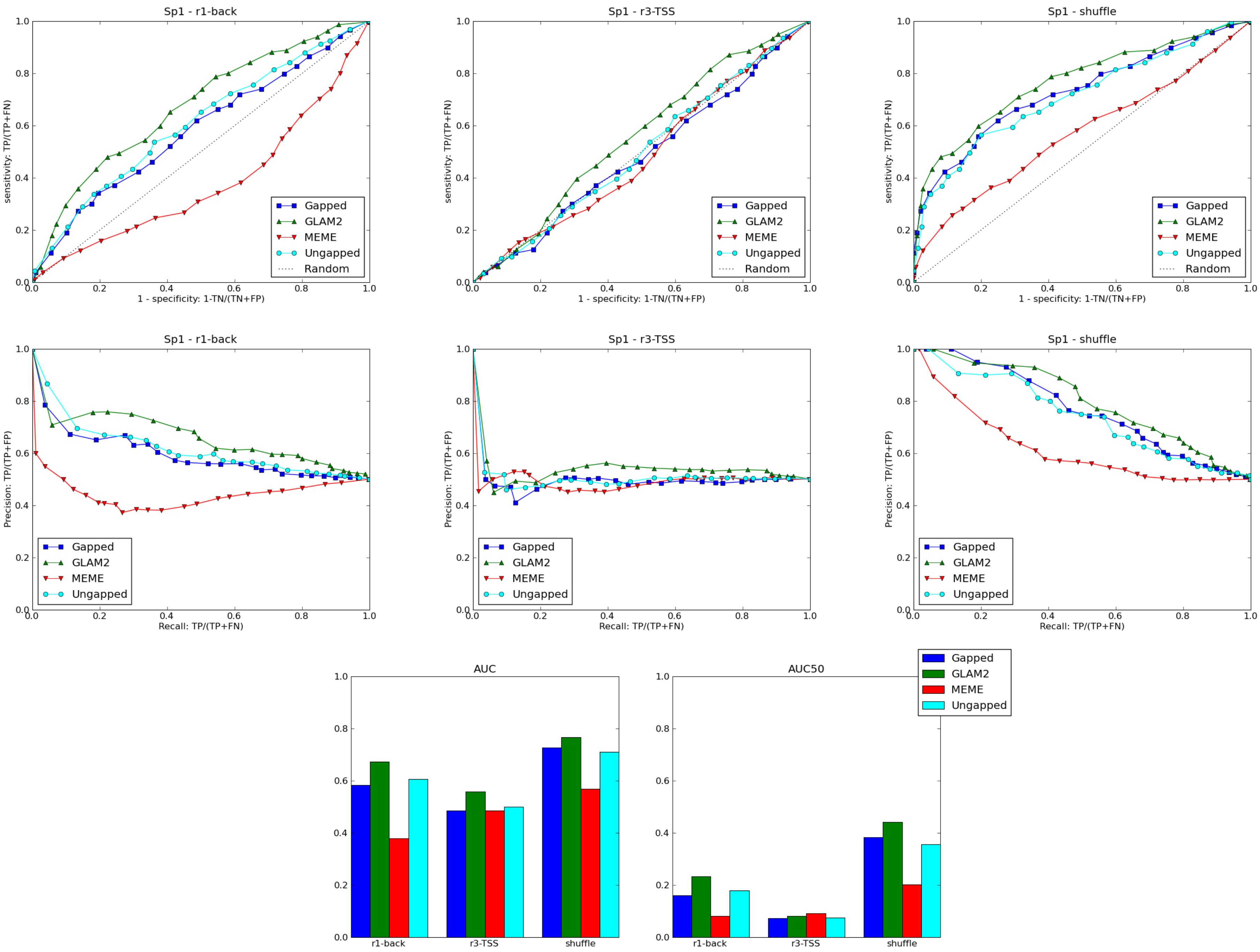
Sp1 - r1-back
Sp1 - r3-TSS
Sp1 - shuffle
sensitivity: TP/(TP+FN)
1 - specificity: 1-TN/(TN+FP)
Precision: TP/(TP+FP)
Recall: TP/(TP+FN)
Gapped
GLAM2
MEME
Ungapped
Random
AUC
AUC50
r1-back
r3-TSS
shuffle

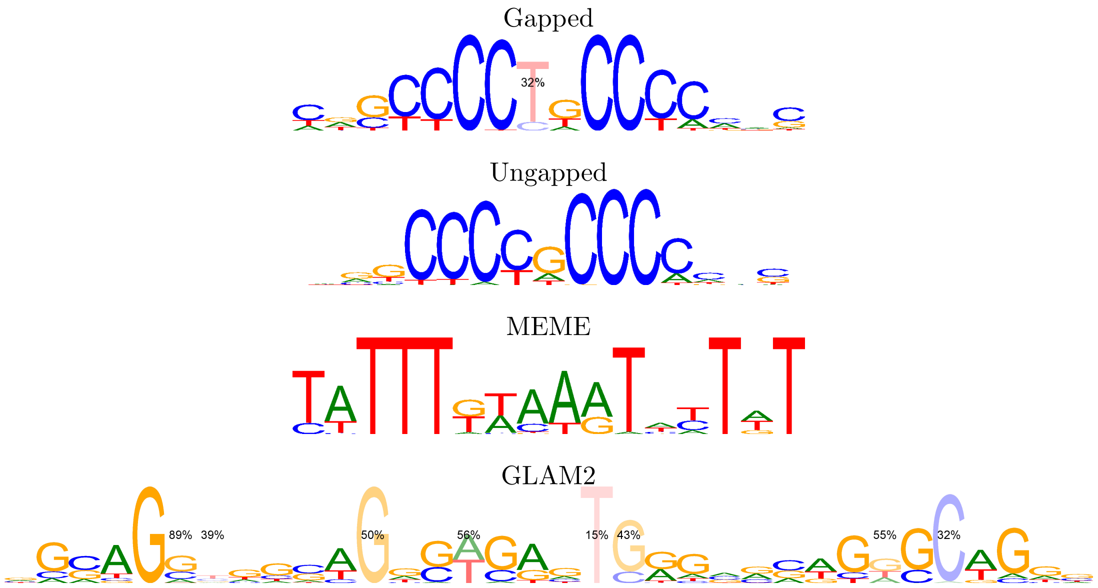
Gapped
32%
Ungapped
MEME
GLAM2
89%
39%
50%
56%
15%
43%
55%
32%

GABP

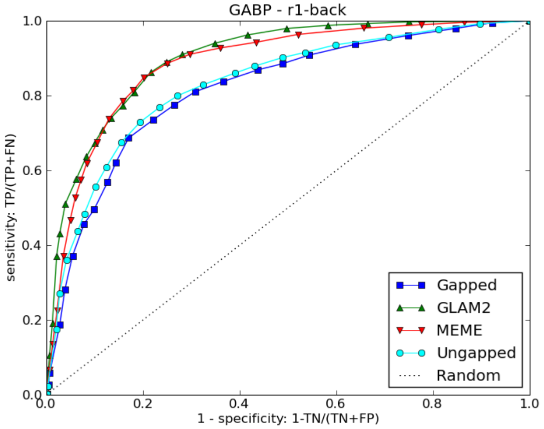
GABP - r1-back
sensitivity: TP/(TP+FN)
1 - specificity: 1-TN/(TN+FP)
Gapped
GLAM2
MEME
Ungapped
Random

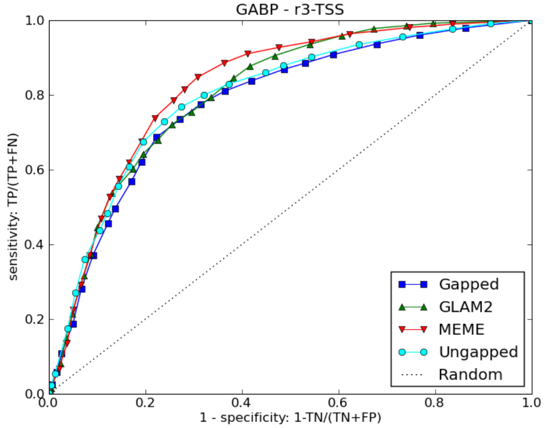
GABP - r3-TSS
sensitivity: TP/(TP+FN)
1 - specificity: 1-TN/(TN+FP)
Gapped
GLAM2
MEME
Ungapped
Random

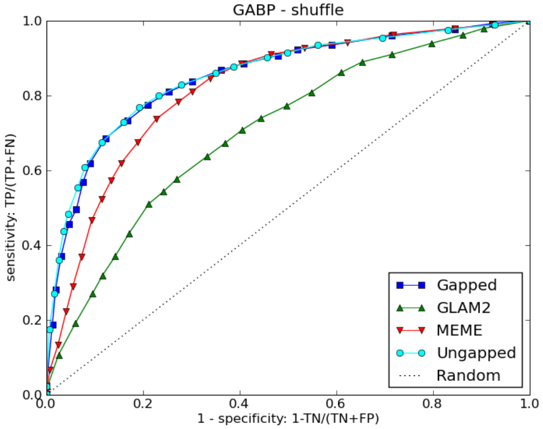
GABP - shuffle
sensitivity: TP/(TP+FN)
1 - specificity: 1-TN/(TN+FP)
Gapped
GLAM2
MEME
Ungapped
Random

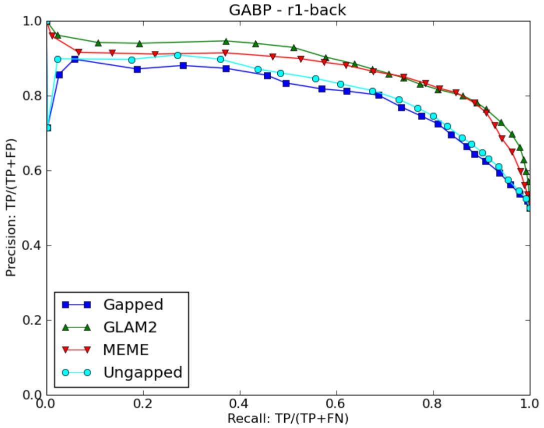
GABP - r1-back
Precision: TP/(TP+FP)
Recall: TP/(TP+FN)
Gapped
GLAM2
MEME
Ungapped

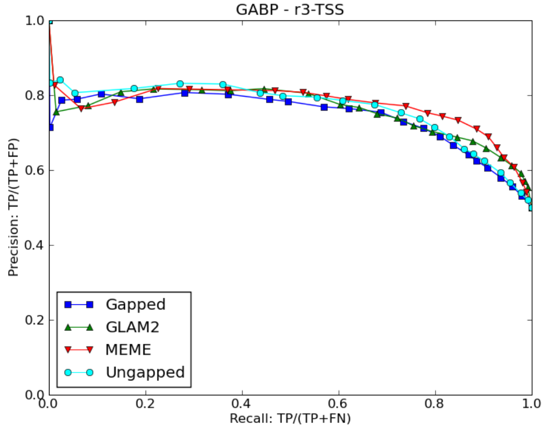
GABP - r3-TSS
Precision: TP/(TP+FP)
Recall: TP/(TP+FN)
Gapped
GLAM2
MEME
Ungapped

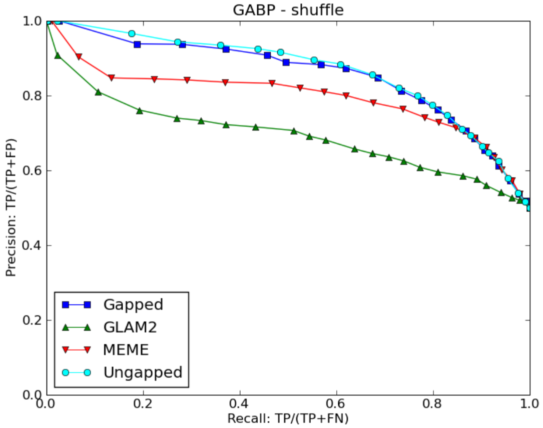
GABP - shuffle
Precision: TP/(TP+FP)
Recall: TP/(TP+FN)
Gapped
GLAM2
MEME
Ungapped

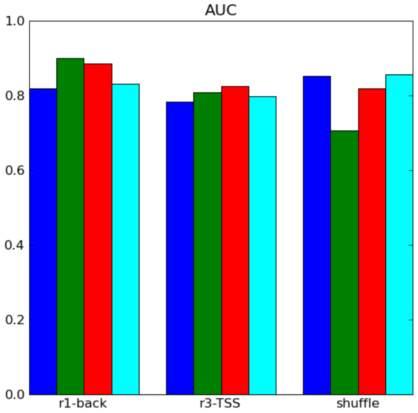
AUC
r1-back
r3-TSS
shuffle

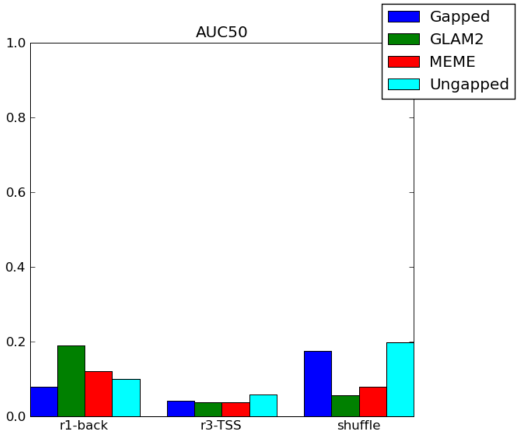
AUC50
r1-back
r3-TSS
shuffle
Gapped
GLAM2
MEME
Ungapped

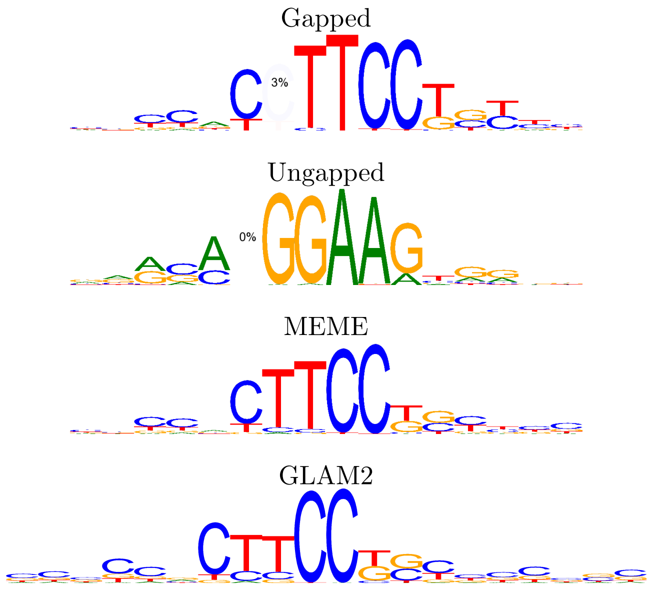

Gapped
3%
Ungapped
0%
MEME
GLAM2

NRSF

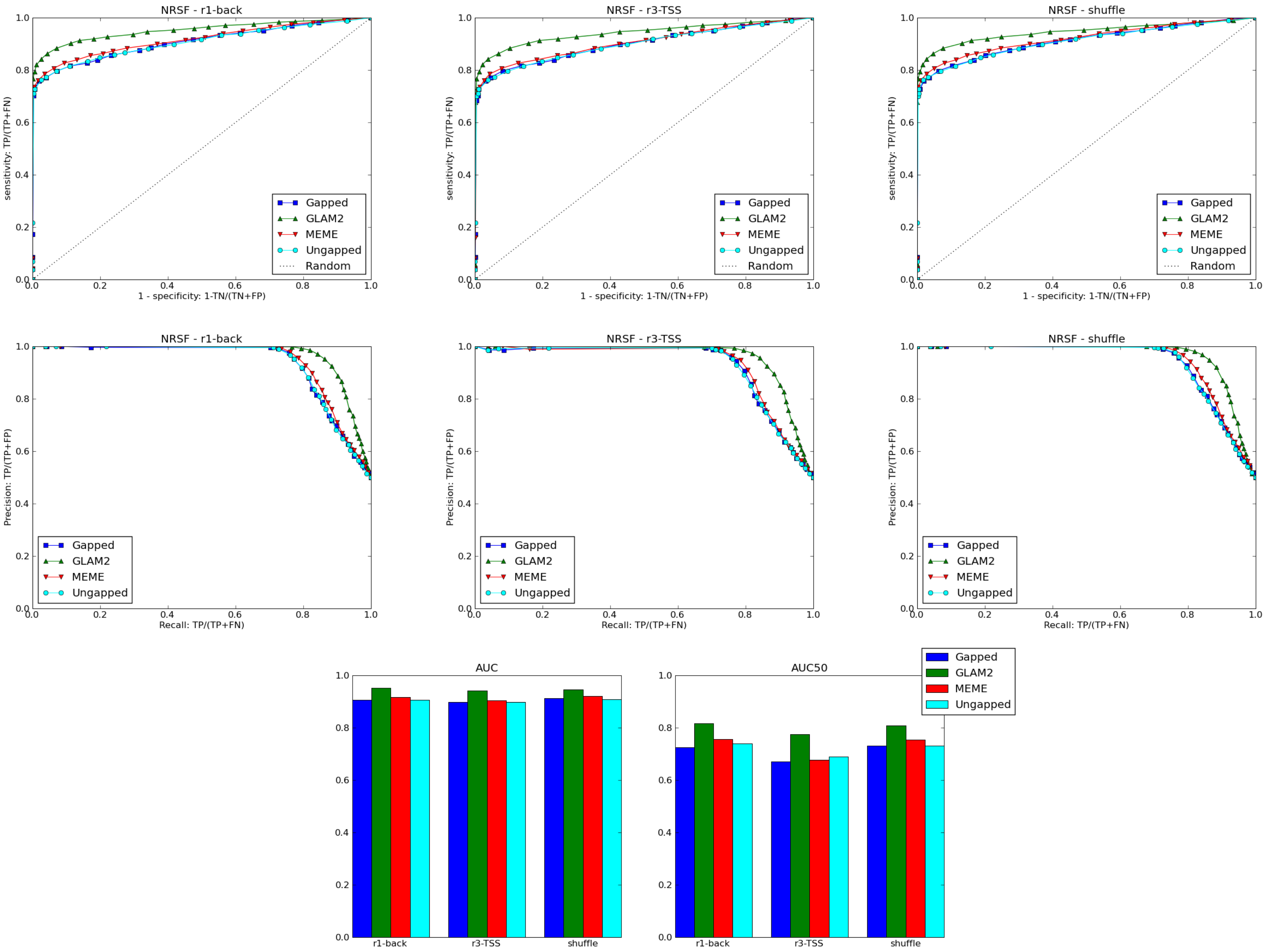

NRSF - r1-back
NRSF - r3-TSS
NRSF - shuffle
sensitivity: TP/(TP+FN)
1 - specificity: 1-TN/(TN+FP)
Precision: TP/(TP+FP)
Recall: TP/(TP+FN)
Gapped
GLAM2
MEME
Ungapped
Random
AUC
AUC50
r1-back
r3-TSS
shuffle

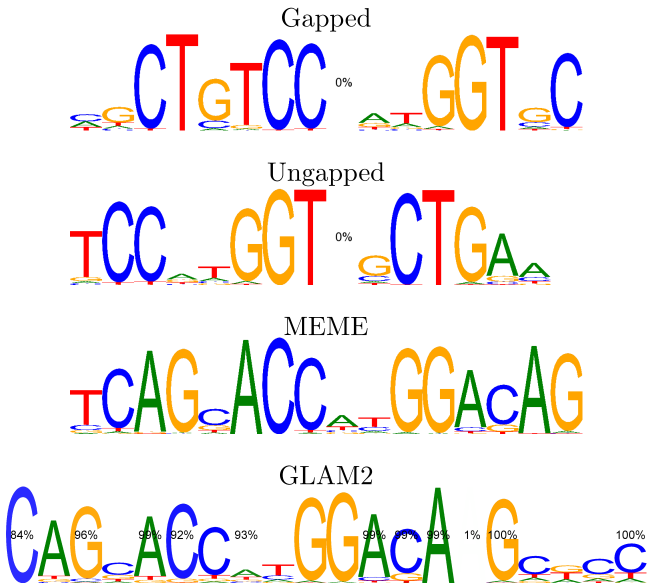
Gapped
0%
Ungapped
0%
MEME
GLAM2
84%
96%
92%
93%
1%
100%
100%

Stat5a

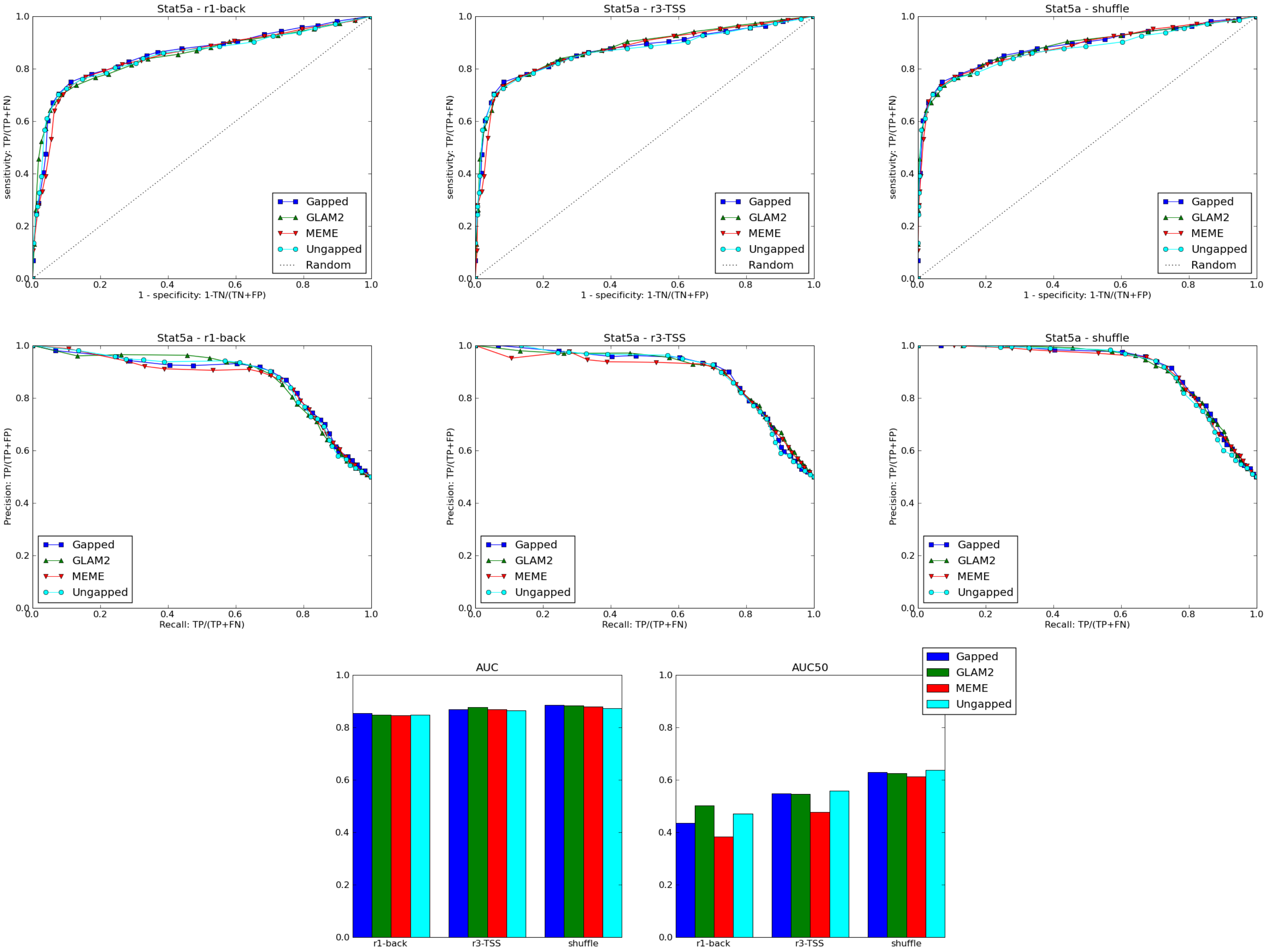

Stat5a - r1-back
Stat5a - r3-TSS
Stat5a - shuffle
sensitivity: TP/(TP+FN)
1 - specificity: 1-TN/(TN+FP)
Precision: TP/(TP+FP)
Recall: TP/(TP+FN)
Gapped
GLAM2
MEME
Ungapped
Random
AUC
AUC50
r1-back
r3-TSS
shuffle

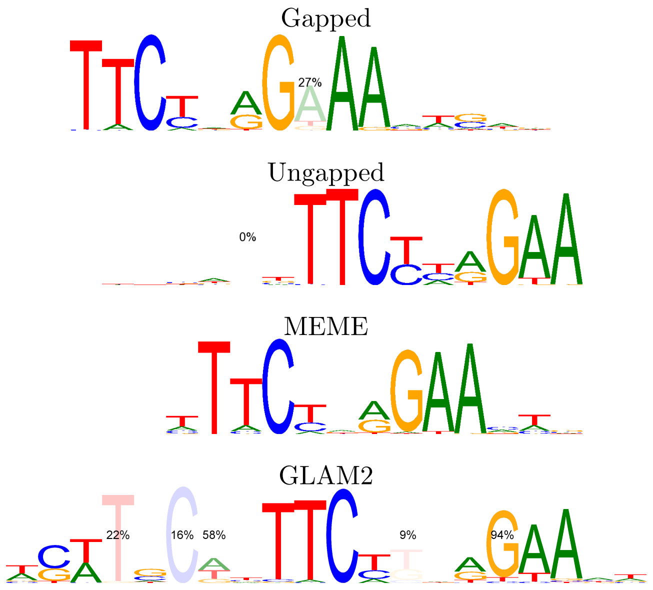
Gapped
27%
Ungapped
0%
MEME
GLAM2
22%
16%
58%
9%
94%

Stat5b

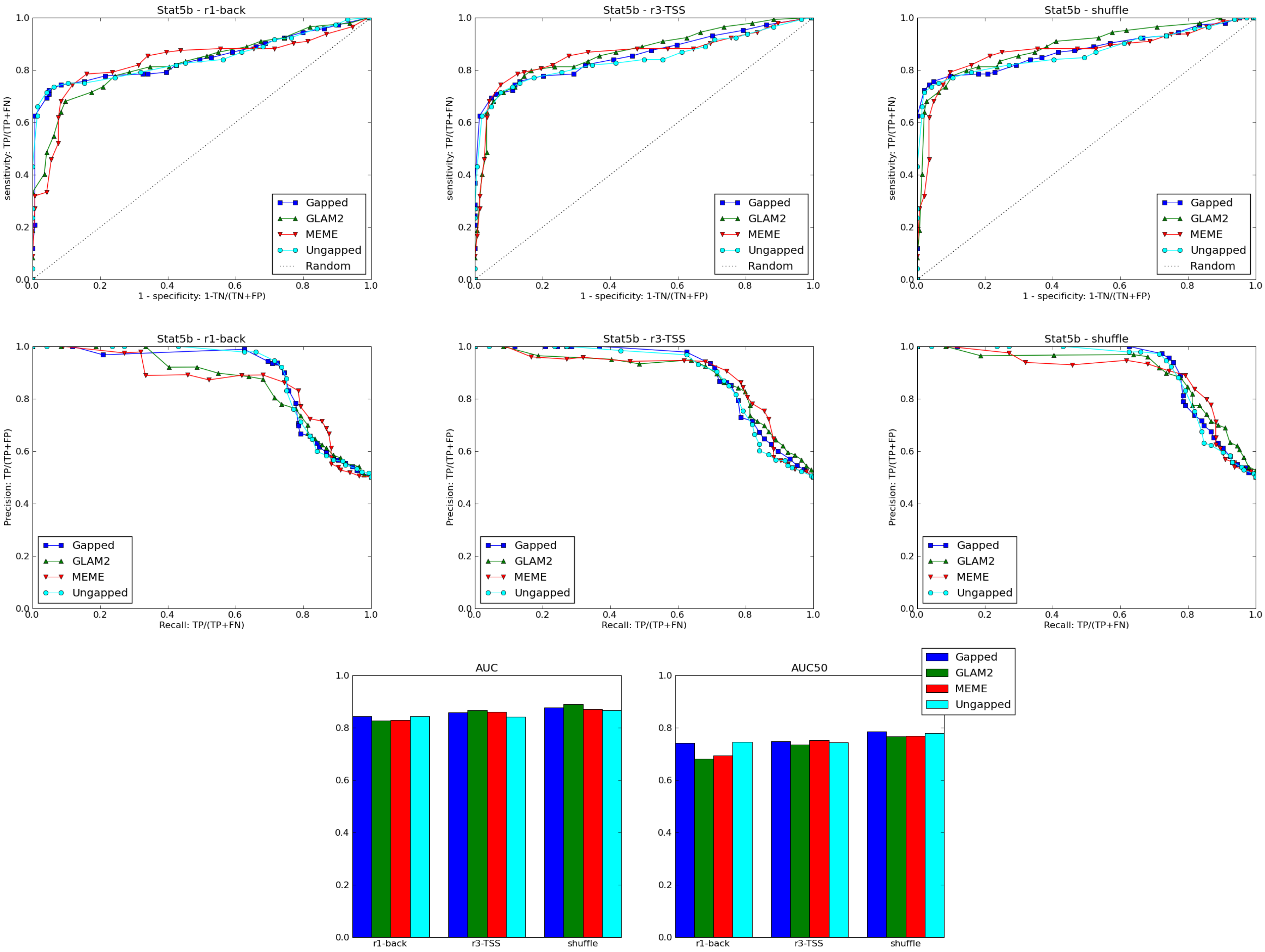

Stat5b - r1-back
Stat5b - r3-TSS
Stat5b - shuffle
sensitivity: TP/(TP+FN)
1 - specificity: 1-TN/(TN+FP)
Precision: TP/(TP+FP)
Recall: TP/(TP+FN)
Gapped
GLAM2
MEME
Ungapped
Random
AUC
AUC50
r1-back
r3-TSS
shuffle
0.0
0.2
0.4
0.6
0.8
1.0

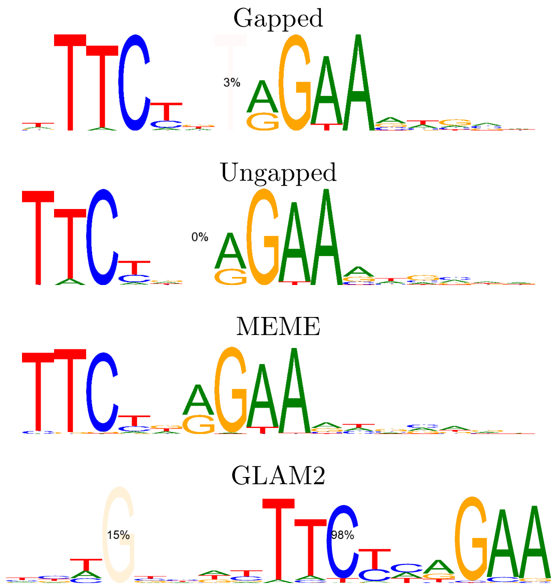
Gapped
3%
Ungapped
0%
MEME
GLAM2
15%
98%

Full Sp1 evaluation

We show ROC curves and precision-recall curves for the full Sp1 evaluation. The AUC statistics are given in the legends of the ROC curves.

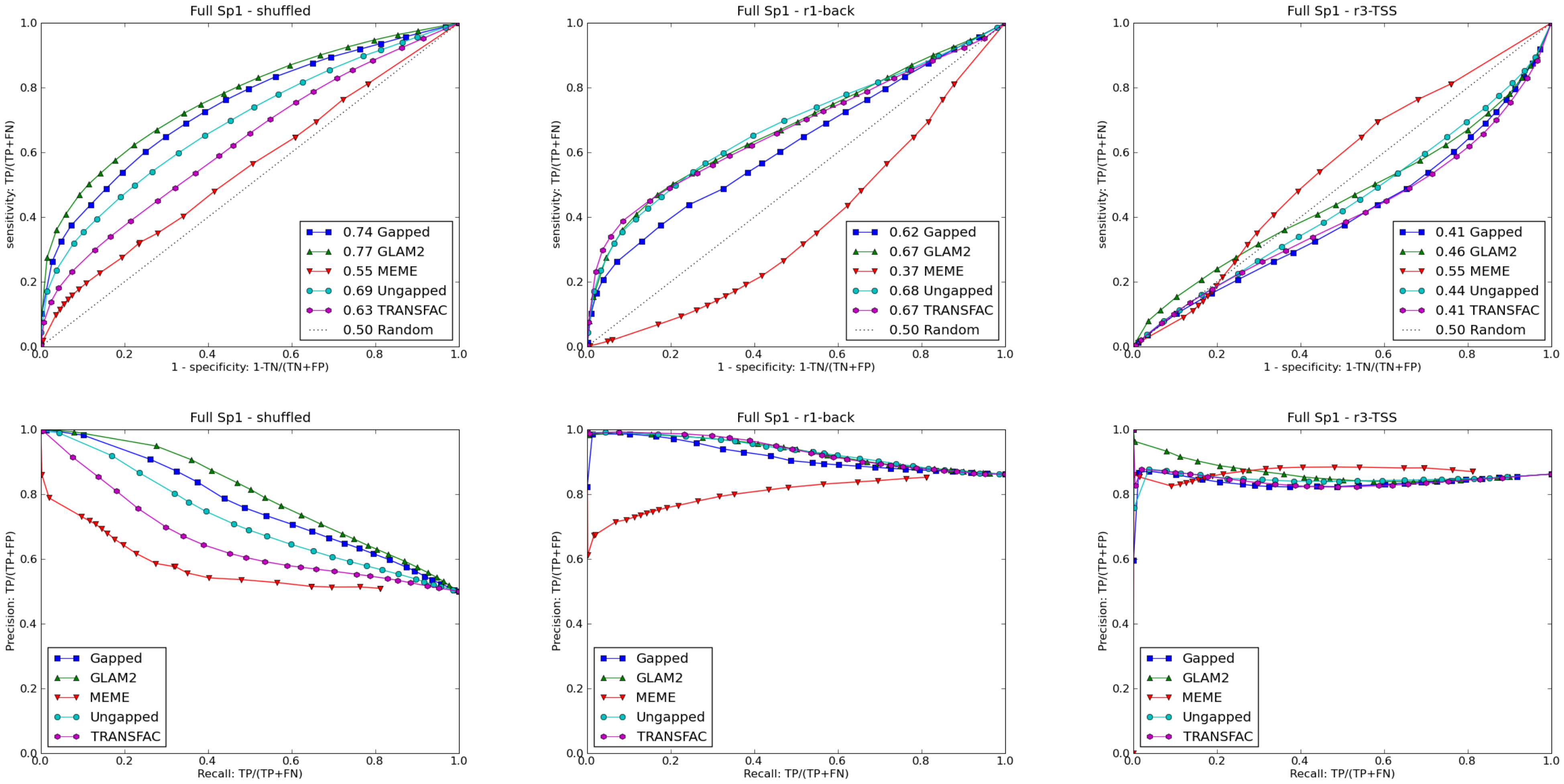

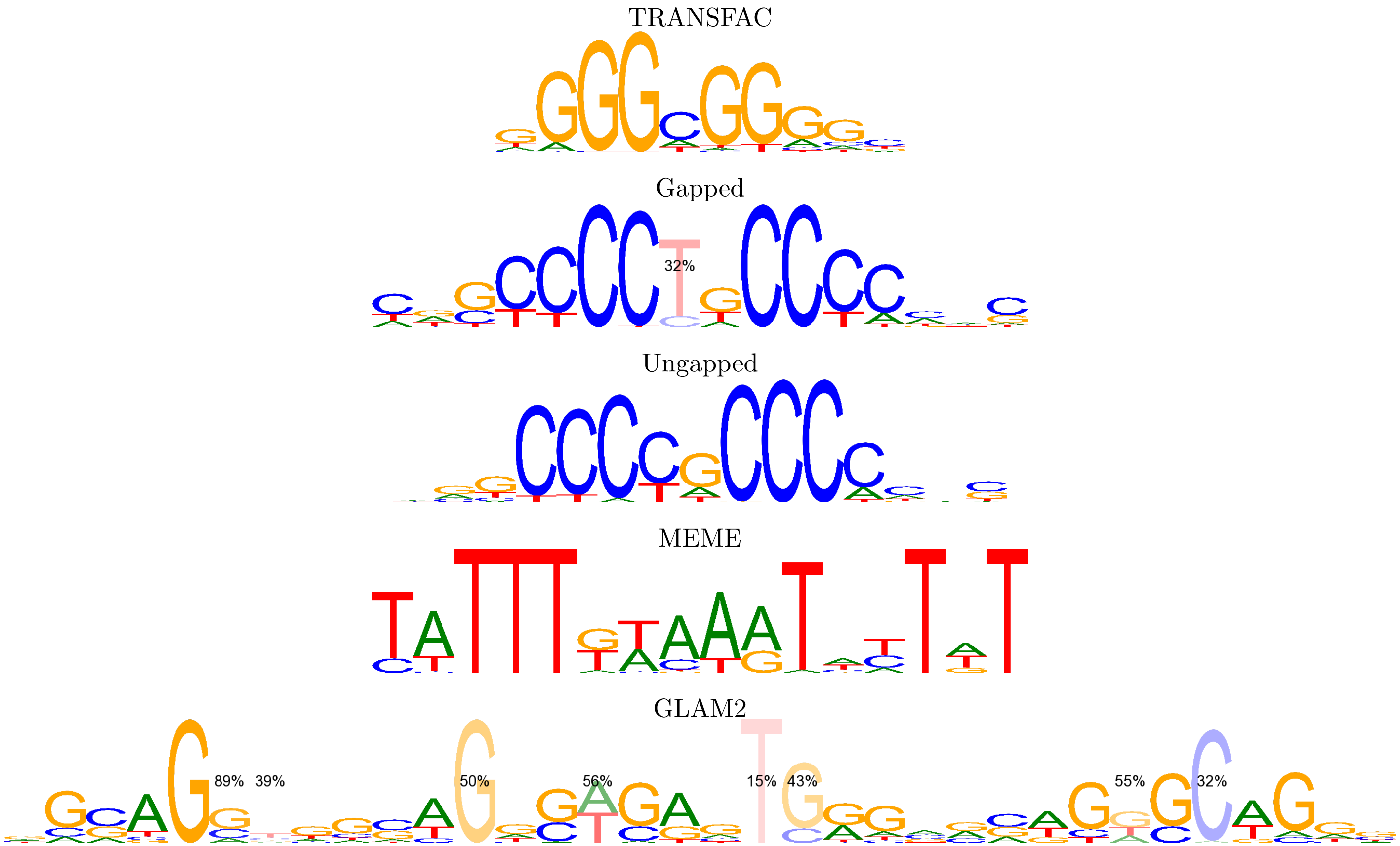
TRANSFAC
Gapped
32%
Ungapped
MEME
GLAM2
89% 39% 50% 56% 15% 43% 55% 32%

RISOTTO

We experimented with RISOTTO [4] (a development of RISO [5]) as it is one of the few methods to allow a variable gap between or within motifs. This method defines parts of motifs by an exact sequence subject to an allowed number of mismatches. This implies an equal weighting to each position in the motif and all or nothing values of the probabilities. This is a much cruder formulation than the PWM formulation used by most work in this area (as discussed in the Background section), and by the standard databases of TF motifs [6, 7]. One of the difficulties of testing the method is the range of parameters that might be given as input: for a given number of mismatches we found that there was a very sharp boundary between the length of motif that gives no models and the motif-length that gives thousands of models. Since the present concern is with the possibility of finding a gapped motif, we report the results where the parameters were set as follows: quorum 75%, boxes 2 (a two part motif), each sub-motif with minimum length 4 bases and maximum length 12 bases, with one substitution (mismatch) allowed in each part; minimum distance between parts 0 bases, maximum distance 3 bases. For p53, the shuffle-algorithm [8] was confused by the N's in the data, but ignoring motifs with N's, the best motif was AAAA_AAAA which was found to be slightly (but not significantly) under represented in the data by the RISOTTO shuffle test. For Sp1, RISOTTO found 7701 models and the best one, AAAA_AGAC, had a Z score of +2.83. For GABP it found 2002 models with the best, AGGA_AGCG, having a Z score of +1.05. Only one model CAGG_GCAG, was found for NRSF but this was found to be under represented by the shuffle test. No models were found for Stat5a. For Stat5b, 208 models were found: the best six models were AACA_CCAG, AACA_ATTT, AACA_TCAG, AACA_GAAG, AACA_TGAT, AAAA_ACTG, and AACA_TCCT—they had Z values ranging from +3.67 to +3.02. Given the number of models, this is of marginal statistical significance, but the decisive point is that these motifs do not correspond to the known motif for this transcription factor. We made various experiments, but at no stage did we have the sense that RISOTTO was a competitive motif-finder for these data sets.

References

[1] Bailey TL, Elkan C: **Fitting a mixture model by expectation maximization to discover motifs in biopolymers**. In *Proceedings of the Second International Conference on Intelligent Systems for Molecular Biology*, AAAI Press 1994:28–36.

[2] Ji H, Jiang H, Ma W, Johnson DS, Myers RM, Wong WH: **An integrated software system for analyzing ChIP-chip and ChIP-seq data.** *Nat Biotechnol* 2008, **26**(11):1293–1300.

[3] Gribskov M, Veretnik S: **Identification of sequence pattern with profile analysis.** *Methods Enzymol* 1996, **266**:198–212.

[4] Pisanti N, Carvalho AM, Carvalho RM, Sagot MF, Marsan L, france Sagot Inesc-id M: **RISOTTO: Fast extraction of motifs with mismatches**. In *Proceedings of the 7th Latin American Theoretical Informatics Symposium, 3887 of LNCS:757-768*, Springer-Verlag 2006:757–768.

[5] Carvalho AM, Freitas AT, Oliveira AL, Sagot MF: **An efficient algorithm for the identification of structured motifs in DNA promoter sequences.** *IEEE/ACM Trans Comput Biol Bioinform* 2006, **3**(2):126–140.

[6] Matys V, Kel-Margoulis OV, Fricke E, Liebich I, Land S, Barre-Dirrie A, Reuter I, Chekmenev D, Krull M, Hornischer K, Voss N, Stegmaier P, Lewicki-Potapov B, Saxel H, Kel AE, Wingender E: **TRANSFAC and its module TRANSCompel: transcriptional gene regulation in eukaryotes.** *Nucleic Acids Res* 2006, **34**(Database issue):D108–D110.

[7] Sandelin A, Alkema W, Engstrom P, Wasserman WW, Lenhard B: **JASPAR: an open-access database for eukaryotic transcription factor binding profiles**. *Nucleic Acids Res* 2004, **32**:D91–D94.

[8] Marsan L, Sagot MF: **Algorithms for extracting structured motifs using a suffix tree with application to promoter and regulatory site consensus identification**. *J. Comput. Bio.* 2000, **7**(3/4):345–360.
