# Supplementary material for: Variable structure motifs for transcription factor binding sites
Source: BMC Genomics. 2010 Jan 14;11:30. doi: 10.1186/1471-2164-11-30 (PMC2824720; doi:10.1186/1471-2164-11-30)
Supplement: Additional file 3 — Application source code. The source code of the implementation of our method. [file 1471-2164-11-30-S3.BZ2 › indexing_suite_v2/docs/indexing_suite_v2.html]

Boost.Python - C++ Container Support

|  |  |
| --- | --- |
|  | Boost.Python  C++ Container Support |

---

## Contents

Introduction

Design goals

Interface
:   container\_suite.hpp

    Container-specific headers

    Using policies

    Visitor flag values

    Extending and customizing
    :   ValueTraits

        ContainerTraits

        Algorithms

        SliceHelper

    Container adapters
    :   container\_proxy

        iterator\_range

    Compiler workarounds

    Known limitations

References

Acknowledgements and Copyright

## Introduction

The purpose of the container indexing suite is to allow Python
code to access C++ containers using regular Python
interfaces. Since each C++ container is different, it is
non-trivial to decide what Python methods can be emulated, and how
to map them to C++ function calls. The indexing suite provides a
framework for representing those decisions, as well as bindings
for the standard C++ container templates. The indexing headers are
in the Boost subdirectory
*boost/python/suite/indexing* and non-template
implementations are in
*libs/python/src/indexing*. Various tests, which can also
serve as examples are in *libs/python/test*.

## Design goals

The primary design goals of the container indexing suite are as
follows. The suite should:

- Support instances of all useful standard container templates
- Provide as much of the normal Python interface as is
  reasonable for each container
- Be extensible to user-defined container types
- Support client-provided CallPolicies

Secondary design goals are as follows. The library should:

- Provide an emulation of Python reference semantics for
  *values* in vector-like containers.
- Provide an emulation of container semantics for iterator
  ranges.

## Interface

The main iterface to the library is via the templated class
`container_suite`, an object of which adds a number
of Python functions to an extension class via a single
`def` call. Support is provided for all of the
standard container templates [1] via
container-specific header files, as shown in the following
example:

```
#include <boost/python/suite/indexing/container_suite.hpp>
#include <boost/python/suite/indexing/vector.hpp>
#include <boost/python/class.hpp>
#include <boost/python/module.hpp>
#include <vector>

BOOST_PYTHON_MODULE(example) {
  class_< std::vector<int> > ("vector_int")
    .def (indexing::container_suite< std::vector<int> >());
}
```

The `container_suite` object achieves this using the
def\_visitor interface, which
provides a hook for the `def` function to install
multiple Python methods in one call. If the container element
type (`int` in the example above) is a user-defined
type, you would have to expose this type to Python via a
separate `class_` instance.

[1] Automatic operation with the standard
containers works properly if your compiler supports partial
template specializations. Otherwise, refer to the compiler workarounds section.

## boost/python/suite/indexing/container\_suite.hpp

The `container_suite.hpp`
header is summarized below:

```
#include <boost/python/suite/indexing/algo_selector.hpp>
#include <boost/python/suite/indexing/visitor.hpp>

#include <boost/python/return_by_value.hpp>
#include <boost/python/return_value_policy.hpp>

namespace boost { namespace python { namespace indexing {
  typedef return_value_policy<return_by_value> default_container_policies;

  template<class Container,
           int Flags = 0,
           class Algorithms = algo_selector<Container> >
  struct container_suite
    : public visitor<Algorithms, default_container_policies, Flags>
  {
    typedef Algorithms algorithms;

    template<typename Policy>
    static visitor<Algorithms, Policy, Flags>
    with_policies (Policy const &policy)
    {
      return visitor <Algorithms, Policy> (policy);
    }
  };
} } }
```

Some important points to note about `container_suite`:

1. It does not include any of the container-specific headers
   (like `vector.hpp` or `set.hpp`), so
   these must be included separately to add support each type
   of container.
2. It derives from the `indexing::visitor`
   template, using a `return_by_value` return
   policy. This is a reasonable default, and follows the
   Boost.Python idiom of passing a default-constructed object
   to the `def` function.
3. The `with_policies` static function template
   generates different instances of the
   `indexing::visitor` template, with
   client-provided policies.
4. The template parameter `Flags` allows client code
   to disable unneeded features in order to reduce code
   size. Details are provided below.

## Container-specific headers

The container indexing suite includes support for many of the
standard C++ container templates, but note that the support code
for each is in a separate header file. These header files (in
the *boost/python/suite/indexing* subdirectory) are:
`vector.hpp`, `deque.hpp`,
`list.hpp`, `set.hpp` and
`map.hpp`. These correspond in the obvious way to the
standard headers `vector`, `deque`,
etc. The header files for the `container_proxy` and `iterator_range` templates
provide their own support implicitly.

## Using policies

You can select call policies using the
`container_suite` static member function
`with_policies` as in the following example:

```
  class_< std::list<heavy_class> > ("list_heavy_class")
    .def (indexing::container_suite< std::list<heavy_class> >
          ::with_policies (my_policies));
```

### Caution with policies

It can be tempting to use `return_internal_reference`
if the container elements are expensive to copy. However, this
can be quite dangerous, since references to the elements can
easily become invalid (e.g. if the element is deleted or
moved). The Boost.Python code for
`return_internal_reference` can only manage the
lifetime of the entire container object, and not those of the
elements actually being referenced. Various alternatives exist,
the best of which is to store the container elements indirectly,
using `boost::shared_ptr` or an equivalent. If this
is not possible,
`container_proxy`
may provide a
solution, at least for vector-like containers.

### Internal policies detail

The `container_suite` object typically adds more than
one function to the Python class, and not all of those functions
can, or should, use exactly the same policies. For instance, the
Python `len` method, if provided, should always
return its result by value. The library actually uses up to
three different sets of policies derived from the one provided
to the `with_policies` function. These are:

1. The supplied policies, unchanged
2. The supplied precall policy only, using `default_call_policies` for result conversion.
3. The supplied precall policies, and the supplied result
   conversion policies applied to *each element* of a
   returned list.

Roughly speaking, methods returning a single container element
use the first option, while methods returning an integer value
(or `void`) use the second option. The third option
applies only to the slice version of `__getitem__`,
which generates a Python list by applying the return conversion
policies to each element in the list.

## Visitor Flag values

The `container_suite` template has an optional
`Flags` parameter that allows client code to disable
various optional features of the suite. This can lead to
significant savings in the size of object files and executables
if features such as sorting or Python slice support are not
needed. The `Flags` parameter (an integer) can be any
bitwise combination of the following values (defined in the
`boost::python::indexing` namespace by `visitor.hpp`):

| Flag | Effect |
| --- | --- |
| `disable_len` | omits the Python `__len__` method |
| `disable_slices` | omits slice support code from `__getitem__`, `__setitem__` and `__delitem__`. |
| `disable_search` | omits the container search methods `count, index` and `__contains__` |
| `disable_reorder` | omits the container reordering operations `sort` and `reverse` |
| `disable_extend` | omits the `extend` method |
| `disable_insert` | omits the `insert` method |

Note that some containers don't support some of the optional
features at all, in which case the relevant flags are
ignored. The value `minimum_support` may be passed as
a flag value to disable all optional features. A simple example
is provided in `test_vector_disable.cpp`

## Extending and customizing

The `container_suite` template relies on seven main
support templates, five of which are suitable for specialization
or replacement by client code. The following diagram shows the
templates [2] and their dependencies, with
the replaceable ones highlighted in grey. For full details,
refer to the specific section on each component – what
follows here is an overview.

|  |
| --- |
|  |
| Diagram 1. Overview of class dependencies |

The `visitor` template, which implements the def\_visitor interface, decides what
Python methods to provide for a container. It takes two template
parameters, `Algorithms` and `Policy` (the
CallPolicies for the Python
methods on the container). The `Algorithms` argument
must provide implementations for the Python methods that the
container supports, as well as a matching
`ContainerTraits` type. This type provides various
compile-time constants that `visitor` uses to decide
what Python features the container provides. It also provides a
`value_traits` typedef, which has similar
compile-time constants related to the values stored in the
container. If the `visitor` instance decides to
provide Python slice support for the container, it instantiates
the `slice_handler` template, which also takes
`Algorithms` and `Policy` parameters. In
such cases, the `Algorithms` argument must supply a
`SliceHelper` type and factory function.

The high-level `container_suite` template uses the
`algo_selector` template to determine what types to
use in the instantiation of `visitor`. The
`algo_selector` template has partial specializations
for all of the STL container templates.

[2] Note that `Algorithms` and
`ContainerTraits` don't represent individual
templates in the diagram, but *groups* of related
templates. For instance, there are actually templates called
`list_algorithms` and `assoc_algorithms`,
among others.

## ValueTraits

A `ValueTraits` class provides simple information
about the type of value stored within a container that will be
exposed to Python via the `container_suite`
interface. It controls the provision of some operations that are
dependant on the operations supported by container elements (for
instance, `find` requires a comparison operator for
the elements). A `ValueTraits` class also provides a
hook called during initialization of the Python class, which can
be used for custom processing at this point.

The following table lists the static constants required in a
`ValueTraits` class:

| Static constant | Type | Meaning |
| --- | --- | --- |
| `equality_comparable` | bool | Whether the value supports comparison via `operator==`. |
| `lessthan_comparable` | bool | Whether the value supports comparison via `operator<`. |

A `ValueTraits` class should provide the following
member function template, which will be called during execution
of the `def` call for the container suite:

```
template <typename PythonClass, typename Policy>
static void visitor_helper (PythonClass &, Policy const &);
```

### Usage notes for ValueTraits

In order to include a custom `ValueTraits` class into
the container suite, it is easiest to supply it as a
specialization of the template
`indexing::value_traits` for the container's element
type. The existing `ContainerTraits` classes all
make use of
`value_traits<container::value_type>`, and so
will use a specialization for the value type if available. The
default, unspecialized, version of `value_traits`
sets both of the static constants to `true` and has
an empty implementation of `visitor_helper`.

As an example, if a user defined type does not have any
comparison operations, then there will probably be compile-time
errors caused by an attempt to provide the Python
`find` or `sort` methods. The solution is
to write a specialized version of
`indexing::value_traits` that disables the
appropriate features. For example:

```
namespace boost { namespace python { namespace indexing {
  template<>
  struct value_traits<my_type> : public value_traits<int>
  {
    static bool const equality_comparable = false;
    static bool const lessthan_comparable = false;
  };
} } }
```

In this example, there is no need to perform any processing in
the `visitor_helper` function, and deriving from an
unspecialized version of the template (e.g.
`value_traits<int>`) exposes an empty
`visitor_helper`.

### Synopsis: boost/python/suite/indexing/value\_traits.hpp

```
namespace boost { namespace python { namespace indexing {
  template<typename T>
  struct value_traits {
    static bool const equality_comparable = true;
    static bool const lessthan_comparable = true;

    template<typename PythonClass, typename Policy>
    static void visitor_helper (PythonClass &, Policy const &)
    { }
  };
} } }
```

## ContainerTraits

A `ContainerTraits` class serves three
purposes. Firstly, it identifies what facilities the container
supports in principle (i.e. either directly or via some support
code). Secondly, it identifies the types used to pass values
into and out of the supported operations. Thirdly, it provides a
hook for additional code to run during initialization of the
Python class (i.e. during the `def` call for the
suite).

Note that a `ContainerTraits` class can be any class,
derived from the existing implementations or not, as long as it
meets the requirements listed in the following sections.

### Static constants for ContainerTraits

The following table lists the static constants that a
`ContainerTraits` class should define. Note that these
must be *compile-time constants*, since parts of the library
use these constants to select between template specializations.
The constants must at least be convertible to the type shown in
the second column.

| Static constant | Type | Meaning | Influence |
| --- | --- | --- | --- |
| `has_copyable_iter` | `bool` | Whether copies of an iterator are independant [3] | Required for `len` and `__iter__` |
| `is_reorderable` | `bool` | Whether it is possible to re-order the contents of the container. | Required for `reverse` and `sort` |
| `has_mutable_ref` | `bool` | Whether container elements can be altered via a reference | Determines `is_reorderable` for most containers. |
| `has_find` | `bool` | Whether find is possible in principle (via member function or otherwise) | `__contains__`, `index`, `count`, `has_key` |
| `has_insert` | `bool` | Whether it is possible to insert new elements into the container. | `insert`, `extend`, slice version of `__setitem__` |
| `has_erase` | `bool` | Whether it is possible to erase elements from the container. | `__delitem__`, slice version of `__setitem__` |
| `has_push_back` | `bool` | Whether container supports insertion at the end. | `append` |
| `has_pop_back` | `bool` | Whether container supports element deletion at the end. | Currently unused |
| `index_style` | `index_style_t` | Type of indexing the container supports [4] | `__getitem__`, `__setitem__`, `__delitem__`, `__iter__`, `extend`, `index`, `count`, `has_key` |

### Notes

|  |  |
| --- | --- |
| [3] | For example, copies of stream iterators are *not* independant. All iterator copies refer to the same stream, which has only one read and one write position. |
| [4] | `index_style_none`, no indexing at all (e.g. `list`)  `index_style_linear`, continuous integer-like index type (e.g. `vector`)  `index_style_nonlinear`, indexing via other types (e.g. `map`). |

### Member types for ContainerTraits

The following table lists the type names that must be defined in
a compatible implementation of `ContainerTraits`.
The large number of types is supposed to provide flexibility for
containers with differing interfaces. For example,
`map` uses the same type for searching and "indexing"
(i.e. `find` and `operator[]`) so
`key_type` and `index_type` would have to
be the same. In contrast, searching a `vector` would
typically use a different type to that used for indexing into a
vector.

| Type name | Meaning |
| --- | --- |
| `container` | The type of the C++ container. |
| `size_type` | The type used to represent the number of elements in the container. |
| `iterator` | The container's iterator type. This should be a non-const iterator unless the container itself is const. |
| `index_type` | The type used to represent indexes extracted from a `__getitem__` call (and others). For `index_style_linear`, this *should be a signed type*, so that negative indices can be processed. For `index_style_nonlinear`, this will most likely be the same type as `key_type`. |
| `index_param` | The type to use when passing `index_type` into a function. |
| `value_type` | The type to use when copying a value into or out of the container. |
| `value_param` | The type to use when passing `value_type` into a function. |
| `key_type` | The type used for search operations like `find` and `count`. |
| `key_param` | The type to use when passing `key_type` into a function. |
| `reference` | The type to use when returning a reference to a container element. |
| `value_traits_` | Traits for the container elements. See the ValueTraits section for information about the requirements on this type. |

### Member functions for ContainerTraits

In order to support additional initialization code to run, a
`ContainerTraits` class should provide a static member
function template as follows:

```
template <typename PythonClass, typename Policy>
static void visitor_helper (PythonClass &, Policy const &);
```

Typically, the implementation would just forward the call to the
equivalent function in the `value_traits_` class.

### Usage notes for ContainerTraits

It may be possible to mix your own `ContainerTraits`
class with one of the existing `Algorithms`
implementations, thus saving yourself a fair bit of work. The
easiest way to do this would be to specialize the
`algo_selector` template for your container type,
using public deriviation to get the implementation from one of
the existing `Algorithms` templates. For example,
assuming that `default_algorithms` is suitable for
your container:

```
namespace boost { namespace python { namespace indexing {
  template<>
  struct algo_selector<my_container>
    : public default_algorithms<my_container_traits>
  {
  };
} } }
```

Alternatively, you could select the algorithms and traits using
the `visitor` template directly, as described in the
compiler workarounds section.

### Simplistic ContainerTraits example

The following block of code shows a simplistic implementation of
`ContainerTraits` for the container
`std::map<std::string, int>`. The actual
implementation used by the suite relies on template
metaprogramming techniques, whereas this example is designed to
show only the essential elements of a
`ContainerTraits` implementation.

```
#include <map>
#include <string>
#include <boost/python/suite/indexing/suite_utils.hpp>
// Include suite_utils to get index_style_t

struct simple_map_traits {
  // Traits information for std::map<std::string, int>

  typedef std::map<std::string, int> container;
  typedef container::size_type       size_type;
  typedef container::iterator        iterator;

  typedef int                        value_type;
  typedef int &                      reference;
  typedef std::string                key_type;
  typedef std::string                index_type;

  typedef int                        value_param;
  typedef std::string const &        key_param;
  typedef std::string const &        index_param;

  static bool const has_copyable_iter = true;
  static bool const has_mutable_ref   = true;
  static bool const has_find          = true;
  static bool const has_insert        = true;
  static bool const has_erase         = true;
  static bool const has_pop_back      = false;
  static bool const has_push_back     = false;
  static bool const is_reorderable    = false;

  static boost::python::indexing::index_style_t const index_style
    = boost::python::indexing::index_style_nonlinear;

  struct value_traits_ {
    // Traits information for our value_type
    static bool const equality_comparable = true;
    static bool const lessthan_comparable = true;
  };

  template<typename PythonClass, typename Policy>
  static void visitor_helper (PythonClass &, Policy const &)
  {
    // Empty
  }
};
```

Example usage of the `simple_map_traits`:

```
#include "simple_map_traits.hpp"

#include <boost/python/suite/indexing/container_suite.hpp>

#include <boost/python/module.hpp>
#include <boost/python/class.hpp>

BOOST_PYTHON_MODULE(test_simple) {
  using namespace boost::python;

  typedef std::map<std::string, int> container_t;
  typedef indexing::map_algorithms<simple_map_traits> algorithms_t;

  class_<container_t> ("map")
    .def (indexing::container_suite<container_t, algorithms_t>());
}
```

## Algorithms

The `Algorithms` requirements are designed to provide
a predictable interface to any container, so that the same
`visitor` code can expose any supported container to
Python. An implemention of `Algorithms` does this by
providing functions and typedefs with fixed names. The exact
interfaces to the functions can vary to some extent, since the
`def` function calls used internally by the
`visitor` deduce the function type
automatically. However, certain points should be confomed to:

1. The functions should be static, with
   `container &` as first parameter.
2. The functions should *not* be overloaded – this
   avoids problems with type deduction.
3. Generally, not all of the possible functions need to be
   implemented, dependant on the static constants in the
   `ContainerTraits`.

The block of code below shows the definition of the
`default_algorithms` class template, which is the
basis for all current implementations of
`Algorithms`. The typedefs that it defines are
primarily for convenience within the implementation itself,
however `container`, `reference` and
`slice_helper` are also required by the
`slice_handler` template, if slices are
supported. Note that `default_algorithms` derives all
of the type information from its `ContainerTraits`
template argument, which allows the same implementation to be
used for various container types.

### Partial boost/python/suite/indexing/algorithms.hpp

```
namespace boost { namespace python { namespace indexing {
  template<typename ContainerTraits, typename Ovr = detail::no_override>
  class default_algorithms
  {
    typedef default_algorithms<ContainerTraits, Ovr> self_type;

  public:
    typedef ContainerTraits container_traits;

    typedef typename ContainerTraits::container   container;
    typedef typename ContainerTraits::iterator    iterator;
    typedef typename ContainerTraits::reference   reference;
    typedef typename ContainerTraits::size_type   size_type;
    typedef typename ContainerTraits::value_type  value_type;
    typedef typename ContainerTraits::value_param value_param;
    typedef typename ContainerTraits::index_param index_param;
    typedef typename ContainerTraits::key_param   key_param;

    typedef int_slice_helper<self_type, integer_slice> slice_helper;

    static size_type size       (container &);
    static iterator  find       (container &, key_param);
    static size_type get_index  (container &, key_param);
    static size_type count      (container &, key_param);
    static bool      contains   (container &, key_param);
    static void      reverse    (container &);
    static reference get        (container &, index_param);
    static void      assign     (container &, index_param, value_param);
    static void      insert     (container &, index_param, value_param);
    static void      erase_one  (container &, index_param);
    static void      erase_range(container &, index_param, index_param);
    static void      push_back  (container &, value_param);
    static void      sort       (container &);

    static slice_helper make_slice_helper (container &c, slice const &);

    template<typename PythonClass, typename Policy>
    static void visitor_helper (PythonClass &, Policy const &);
  };
} } }
```

### Slice support

For containers that support Python slices, the
`visitor` template will instantiate and use
internally the `slice_handler` template. This
template requires a type called `slice_helper` and a
factory function called `make_slice_helper` from its
`Algorithms` argument. More details are provided in
the section SliceHelper.

### Usage notes for Algorithms

The existing `indexing::algo_selector` template uses
partial specializations and public derivation to select an
`Algorithms` implementation suitable for any of the
standard container types. Exactly how it does this should be
considered an implementation detail, and uses some tricks to
reuse various existing `Algorithms`
implementations. In any case, client code can specialize the
`algo_selector` template for new container types, as
long as the specialized instances conform to the requirements
for `Algorithms` as already given.

A new implementation of `Algorithms` could derive
from any one of the existing implementation templates, or be
completely independant. The existing implementation templates
are listed in the following table. They each take one template
parameter, which should be a valid `ContainerTraits`
class, as specified in a previous
section.

| Template name | Description |
| --- | --- |
| `default_algorithms` | Uses standard iterator-based algorithms wherever possible. Assumes that the container provides `begin` and end `end` member functions that return iterators, and some or all of `size`, `insert`, `erase` and `push_back`, depending on what functions get instantiated. |
| `list_algorithms` | Similar to the above (in fact, it derives from `default_algorithms`) except that it uses container member functions `reverse` and `sort` instead of the iterator-based versions. Defined in `boost/python/suite/indexing/list.hpp`. |
| `assoc_algorithms` | Also derived from `default_algorithms`, for use with associative containers. Uses the container member function `find` for indexing, and member function `count` instead of iterator-based implementations. |
| `set_algorithms` | Derived from `assoc_algorithms` to handle `set` insertion operations, which are slightly different to the `map` versions. |
| `map_algorithms` | Derived from `assoc_algorithms` to handle `map` insertion and lookup, which are slightly different to the `set` versions. |

The `default_algorithms` template attempts to place
as few restrictions as possible on the container type, by using
iterators and standard algorithms in most of its functions. It
accepts an optional second template parameter, which can be used
via the curiously recurring template idiom to replace any of its
functions that it relies on internally. For instance, if you've
created an iterator-style interface to a container that is not
at all STL-like (let's call it `weird_container`),
you might be able to re-use most of
`default_algorithms` by replacing its basic functions
like this:

```
namespace indexing = boost::python::indexing;

struct my_algorithms
  : public indexing::default_algorithms <
      weird_container_traits, my_algorithms
  >
{
  size_t size (weird_container const &c) {
    return ...;
  }

  my_iterator_t begin (weird_container &c) {
    return ...;
  }

  my_iterator_t end (weird_container &c) {
    return ...;
  }
};
```

## SliceHelper

Support code for Python slices is split into two portions, the
`slice_handler` template, and a "slice helper" that
can easily be replaced by client code via a typedef and factory
function in the `Algorithms` argument supplied to
`container_suite`. The slice helper object takes care
of reading and writing elements from a slice in a C++ container,
and optionally insertion and deletion. Effectively, the slice
helper object maintains a pointer to the current element of the
slice within the container, and provides a `next`
function to advance to the next element of the slice. The
container suite uses the following interface for slices:

| Expression | Return type | Notes |
| --- | --- | --- |
| `Algorithms::` `make_slice_helper` `(c, s)` | `Algorithms::` `slice_helper` | `c` is of type `Algorithms::` `container &` and `s` is of type `indexing::` `slice const &`. Returns a newly constructed `slice_helper` object by value. |
| `slice_helper.``next()` | `bool` | Advances the slice helper's current element pointer to the next element of the slice. Returns true if such an element exists, and false otherwise. The first time this function is called, it should set the current pointer to the first element of the slice (if any). |
| `slice_helper.` `current()` | `Algorithms::` `reference` | Returns a reference to the current element of the slice. This will only be called if the last call to `next()` returned true. |
| `slice_helper.``write (v)` | `void` | The parameter `v` is of type `Algorthims::value_param`. Advances to the next element of the slice, as defined in `next`, and writes the given value `v` at the new location in the container.If the slice is exhausted (i.e. `next` would return false) then `write` *either* inserts the value into the container at the next location (past the end of the slice), *or* sets a Python exception and throws. |
| `slice_helper.` `erase_remaining()` | `void` | *Either* erases any remaining elements in the slice not already consumed by calls to `next` or `write`, *or* sets a Python exception and throws. |

The container suite provides a generic implementation of the
`SliceHelper` requirements for containers that have
integer-like indexes. It is parameterized with a
`SliceType` parameter that allows the integer index
values to come from various different sources, the default being
the `PySlice_GetIndices` function. Refer to the
header file `int_slice_helper.hpp`
and the references to it in the `algorithms.hpp`
header for details.

## container\_proxy

The `container_proxy` template provides an emulation
of Python reference semantics for objects held by value in a
vector-like container. Of course, this introduces some
performance penalties in terms of memory usage and run time, so
the primary application of this template is in situations where
all of the following apply:

1. It is not practical to switch to a container of shared
   pointers
2. Python code requires reference semantics for the objects
   within the container
3. Element insertion, deletion or assignment takes place, so
   that using `return_internal_reference` would be
   dangerous.

The `container_proxy` template wraps any vector-like
container and presents an interface that is similar to that of
`std::vector`, but which returns
`element_proxy` objects instead of plain references
to values stored in the wrapped container. During an operation
that alters the position of an element within the container
(e.g. `insert`) the `container_proxy` code
updates the relevant proxy objects, so that they still refer to
the *same* elements at their new locations. Any operation
that would delete or overwrite a value in the container
(e.g. `erase`) copies the to-be-deleted value into
its corresponding proxy object. This means that a proxy's
"reference" to an element is robust in the face of changes to
the element's position in the container, and even the element's
removal.

Ideally, any code that changes the positions of elements within
the container would use only the `container_proxy`
interface, to ensure that the proxies are maintained in
synchronization. Code that otherwise makes direct modifications
to the raw container must notify the
`container_proxy` of the changes, as detailed in the
following section.

### container\_proxy interface

The `container_proxy` template takes three
parameters, only the first of which is mandatory:

```
template<class Container
       , class Holder = identity<Container>
       , class Generator = vector_generator> class container_proxy;
```

The `Container` argument is the raw container type
that the `container_proxy` will manage. It must
provide random-access indexing.

The `Holder` argument determines how the
`container_proxy` stores the raw container object.
There are currently two types of holder implemented, the default
`identity` template which will store the raw
container by value within the `container_proxy`, and
the `deref` template which stores a (plain) pointer
to an external object. It would also be possible, for instance,
to create a holder that uses a `shared_pointer`, or
one that stores a pointer but performs deep copies.

The `Generator` argument determines what container to
use for storing the proxy objects. The argument must be a
suitable class so that
`Generator::apply<proxy_t>::type` is a typedef
for the container to use for storing the proxies. The default is
`vector_generator`, which generates
`std::vector` instances. The usefulness of allowing
other generators can be seen from the example
`container_proxy<std::deque<...> >`.
Insertion at the beginning of this `container_proxy`
requires an insertion at the beginning of the
`std::deque` raw container, which has amortized
constant time complexity. However, it also requires an insertion
at the beginning of the proxy container, which (using the
`std::vector` provided by
`vector_generator`) has linear time complexity. If
this is a significant issue, you can use a custom
`Generator` to match the performance characteristics
of the proxy container to those of the raw container.

Examples in libs/python/test/test\_container\_proxy.cpp
...

### Synopsis: boost/python/suite/indexing/container\_proxy.hpp

```
namespace boost { namespace python { namespace indexing {
  template<class Container
         , class Holder = identity<Container>
         , class Generator = vector_generator>
  class container_proxy
  {
  public:
    typedef typename Container::size_type size_type;
    typedef typename Container::difference_type difference_type;
    typedef typename Container::value_type raw_value_type;

    typedef typename Holder::held_type held_type;

    typedef implementation defined value_type;
    typedef implementation defined const_value_type;
    typedef implementation defined iterator;
    typedef implementation defined const_iterator;

    typedef value_type        reference;       // Has reference semantics
    typedef const_value_type  const_reference; // Has reference semantics

    container_proxy ();
    explicit container_proxy (held_type const &);
    template<typename Iter> container_proxy (Iter, Iter);

    container_proxy (container_proxy const &);
    container_proxy &operator= (container_proxy const &);
    ~container_proxy ();

    Container const &raw_container() const;   // OK to expose const reference

    reference       at (size_type);
    const_reference at (size_type) const;

    reference       operator[] (size_type);
    const_reference operator[] (size_type) const;

    size_type size() const;
    size_type capacity() const;
    void reserve(size_type);

    iterator begin();
    iterator end();

    iterator erase (iterator);
    iterator erase (iterator, iterator);
    iterator insert (iterator, raw_value_type const &);
    template<typename Iter> void insert (iterator, Iter, Iter);

    void push_back (raw_value_type const &);
    value_type pop_back ();

    // These functions are not normally necessary. They notify the
    // container_proxy of changes to the raw container made by other
    // code (see documentation for details)
    void detach_proxy (size_type index);
    void detach_proxies (size_type from, size_type to);
    void prepare_erase (size_type from, size_type to);
    void notify_insertion (size_type from, size_type to);
  };
} } }
```

The `identity` template.

```
namespace boost { namespace python { namespace indexing {
  template<typename T> struct identity {
    typedef T held_type;

    static T &       get(T &       obj) { return obj; }
    static T const & get(T const & obj) { return obj; }

    static T    create ()                     { return T(); }
    static T    copy   (T const &copy)        { return copy; }
    static void assign (T &to, T const &from) { to = from; }
    static void pre_destruction (T &)         { }
  };
} } }
```

The `deref` template.

```
namespace boost { namespace python { namespace indexing {
  template<typename P> struct deref {
    typedef P held_type;

    typedef typename boost::iterator_value<P>::type     value;

    static value &       get (P &       ptr)  { return *ptr; }
    static value const & get (P const & ptr)  { return *ptr; }

    static P    create ()                     { return P(); }
    static P    copy   (P const &copy)        { return copy; }
    static void assign (P &to, P const &from) { to = from; }
    static void pre_destruction (P &)         { }
  };
} } }
```

The `vector_generator` class.

```
namespace boost { namespace python { namespace indexing {
  struct vector_generator {
    template<typename Element> struct apply {
      typedef std::vector<Element> type;
    };
  };
} } }
```

### container\_proxy implementation notes

An `element_proxy` refers to an element of the
container via two levels of indirection – it holds a
pointer to a so-called `shared_proxy` object, which
has a pointer back to the `container_proxy` object
and an element index within the wrapped container. This can be
seen in the following diagram, which shows a
`container_proxy< vector<int> >`
containing the three elements 111, 222 and 333.

|  |
| --- |
|  |
| Diagram 2. Example of `container_proxy` with an element proxy |

In the example above, the shown `element_proxy`
object refers (indirectly) to the container element with the
value 222. An insertion before this element would increment the
index numbers in the `shared_proxy` objects so that
the given `element_proxy` continues to refer to the
same value at its new location. Similary, a deletion before the
element would decrement the affected `shared_proxy`
indexes. If the referenced element itself gets deleted or
overwritten, the `shared_proxy` first takes a
*copy* of the original value, and is then considered to be
*detached* from the `container_proxy`. This
situation is shown below in diagram 3.

|  |
| --- |
|  |
| Diagram 3. Example of `element_proxy` with detached `shared_proxy` |

## iterator\_range

The `iterator_range` template provides a
container-like interface to a range defined by two iterators.
The interface is complete enough to provide any Python method
that does not require insertion or deletion, e.g.
`len`, `index` and `sort`. See
the `get_array_plain` function in libs/python/test/test\_array\_ext.cpp
for an example usage. If you only need iteration over the values
in a range, consider using the simpler `range`
function provided by boost/python/iterator.hpp

Beware that C++ iterators are not very Python-like, since they
do not provide any guarantees about the lifetimes of the objects
they refer to. Invalidating either of the iterators stored in an
`iterator_range` object is dangerous, since
subsequently using the iterators (from Python or C++) results in
undefined behaviour.

`iterator_range` should work with any
`ForwardIterator` type.

### Synopsis: boost/python/suite/indexing/iterator\_range.hpp

```
namespace boost { namespace python { namespace indexing {
  template<typename Iterator>
  class iterator_range
  {
  private:
    typedef typename boost::call_traits<Iterator>::param_type iterator_param;
    typedef std::iterator_traits<Iterator> std_traits;

  public:
    typedef typename std_traits::reference       reference;
    typedef Iterator                             iterator;
    typedef typename std_traits::difference_type size_type;
    typedef typename std_traits::difference_type difference_type;
    typedef typename std_traits::value_type      value_type;
    typedef typename std_traits::pointer         pointer;

    iterator_range (iterator_param, iterator_param);
    iterator_range (std::pair<iterator, iterator> const &);

    iterator begin() const;
    iterator end() const;

    size_type size () const;
    reference operator[] (size_type) const;
    reference at (size_type) const;

  private:
    // Member variables
  };

  template<typename T, std::size_t N> T *begin (T (&array)[N]);
  template<typename T, std::size_t N> T *end   (T (&array)[N]);

} } }
```

## Compiler workarounds

It is possible to use the suite without partial template
specialization support, but the `algo_selector`
specializations for the standard containers does not work. To
avoid this problem, the client code must explicitly select the
`Algorithms` and `ContainerTraits`
instances to be used, and there are some additional support
templates in the container-specific header files for this
purpose.

```
#include <boost/python/suite/indexing/vector.hpp>

...

  using namespace boost::python;
  using namespace boost::python::indexing;

  class_<std::vector<int> > ("vector_int")
    .def (indexing::vector_suite <vector <int> >());
```

Microsoft Visual C++ 6.0 has a version of the standard
`deque` header that is incompatible with the
`container_proxy` template, since it lacks a correct
template version of the `insert` member function. An
updated copy of the `deque` header that fixes this
problem (among others) is available directly from Dinkumware
(at time of writing, 2003/11/04).

## Known limitations

This section lists known limitations of the container
interfaces. These may or may not get fixed in the future, so
check the latest release of Boost and/or the Boost CVS
repository. Feel free to submit your own improvements to the
mailing list for the Python C++-SIG.

The following Python sequence and mapping functions are not
currently implemented for any containers:
`keys, values, items, clear, copy, update,`
`pop, __add__, __radd__, __iadd__, __mul__, __rmul__`
and `__imul__`.
Most of the methods mentioned (except for `pop`)
present no particular difficulty to implement. The problem with
`pop` is that it is incompatible with some return
value policies (for instance,
`return_internal_reference`) since it must return a
copy of an element that has already been removed from the
container. This probably requires an extension to the
`container_suite` interface, to allow the client code
the option of specifying a different return policy for this
method in particular.

The suite currently restricts itself to the normal Python
container interface methods, which do not expose all of the
interfaces available with the C++ containers. For example,
vector `reserve` has no equivalent in Python and is
not exposed by the suite. Of course, user code can still add a
`def` call for this manually.

The `map` iterator should return only the key part of
the values, but currently returns the whole
`std::pair`.

The `sort` method (where provided) should allow an
optional comparison function from Python.

## References

The Python Library Reference section on Sequence
Types and the Python Reference Manual section on Emulating
container types. The C++
Standard.

## Acknowledgements and Copyright

Thanks to Joel de Guzman and David Abrahams for input and
encouragement during the development of the container suite, and
to and Ralf W. Grosse-Kunstleve for his invaluable support in
porting to various platforms. Joel wrote the original
implementation of the indexing support, which provided many of
the ideas embodied in the new implementation.

The container suite code and documentation are Copyright (c)
2003 by Raoul Gough, and licensed according to the Boost license.
