# Supplementary figures and images for: Variable structure motifs for transcription factor binding sites
Source: BMC Genomics. 2010 Jan 14;11:30. doi: 10.1186/1471-2164-11-30 (PMC2824720; doi:10.1186/1471-2164-11-30)

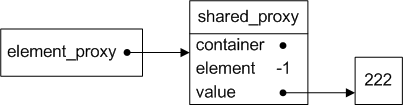

Supplement: Additional file 3 — Application source code. The source code of the implementation of our method. [file 1471-2164-11-30-S3.BZ2 › indexing_suite_v2/docs/indexing_suite_v2_files/proxy_detached.png]

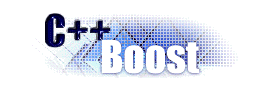

Supplement: Additional file 3 — Application source code. The source code of the implementation of our method. [file 1471-2164-11-30-S3.BZ2 › indexing_suite_v2/docs/indexing_suite_v2_files/cboost.gif]

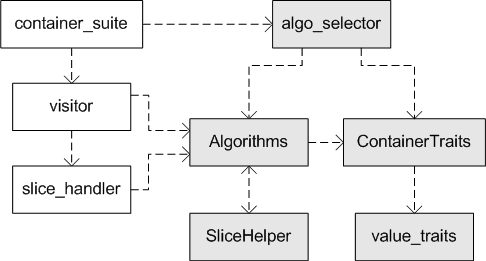

Supplement: Additional file 3 — Application source code. The source code of the implementation of our method. [file 1471-2164-11-30-S3.BZ2 › indexing_suite_v2/docs/indexing_suite_v2_files/overview.png]

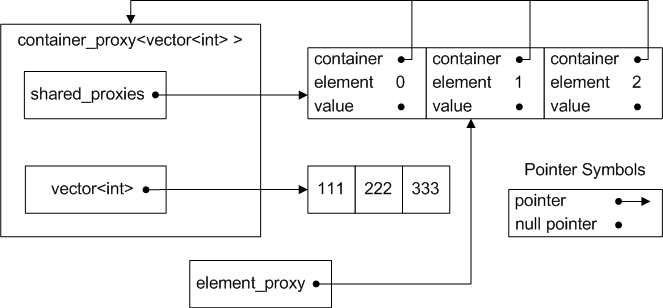

Supplement: Additional file 3 — Application source code. The source code of the implementation of our method. [file 1471-2164-11-30-S3.BZ2 › indexing_suite_v2/docs/indexing_suite_v2_files/proxy.png]
