# Supplementary material for: Variable structure motifs for transcription factor binding sites
Source: BMC Genomics. 2010 Jan 14;11:30. doi: 10.1186/1471-2164-11-30 (PMC2824720; doi:10.1186/1471-2164-11-30)
Supplement: Additional file 3 — Application source code. The source code of the implementation of our method. [file 1471-2164-11-30-S3.BZ2 › Infpy/python/infpy/dp/doc/mvn.pdf]

$$f(x) = \frac{1}{(2\pi)^{N/2}|\Sigma|^{1/2}} \exp(-\frac{1}{2}(x-\mu)^T\Sigma^{-1}(x-\mu))$$
